# Substrates mimicking the blastocyst geometry revert pluripotent stem cell to naivety

**DOI:** 10.1038/s41563-024-01971-4

**Published:** 2024-08-12

**Authors:** Xun Xu, Weiwei Wang, Yue Liu, Johan Bäckemo, Matthias Heuchel, Wei Wang, Yan Nie, Imran Iqbal, Karl Kratz, Andreas Lendlein, Nan Ma

**Affiliations:** 1 Institute of Active Polymers and Berlin-Brandenburg Center for Regenerative Therapies, Helmholtz-Zentrum Hereon, Teltow, Germany; 2 Institute of Chemistry, University of Potsdam, Potsdam, Germany; 3 Helmholtz Virtual Institute—Multifunctional Biomaterials for Medicine, Berlin and Teltow, Teltow, Germany; 4 Institute of Chemistry and Biochemistry, Freie Universität Berlin, Berlin, Germany

**Keywords:** Biomaterials - cells, Biomaterials - cells

## Abstract

Naive pluripotent stem cells have the highest developmental potential but their in vivo existence in the blastocyst is transient. Here we report a blastocyst motif substrate for the in vitro reversion of mouse and human pluripotent stem cells to a naive state. The substrate features randomly varied microstructures, which we call motifs, mimicking the geometry of the blastocyst. Motifs representing mouse-blastocyst-scaled curvature ranging between 15 and 62 mm^−1^ were the most efficient in promoting reversion to naivety, as determined by time-resolved correlative analysis. In these substrates, apical constriction enhances E-cadherin/RAC1 signalling and activates the mechanosensitive nuclear transducer YAP, promoting the histone modification of pluripotency genes. This results in enhanced levels of pluripotency transcription factor NANOG, which persist even after cells are removed from the substrate. Pluripotent stem cells cultured in blastocyst motif substrates display a higher development potential in generating embryoid bodies and teratomas. These findings shed light on naivety-promoting substrate design and their large-scale implementation.

## Abstract (web summary)

Substrates with blastocyst-like microstructures effectively revert primed pluripotent stem cells to naivety by promoting cell–cell apical constriction and mechanoinduced epigenetic activation of naive pluripotency genes.

## Main

In nature, naive and primed states of pluripotent stem cells emerge sequentially in pre- and post-implantation embryos^1,2^. These states are distinct in morphology, clonogenic capacity, transcriptome and epigenetic signatures^3^. Naive pluripotent stem cells (nPSCs) have shown robust proliferation and enhanced directed differentiation^4^, especially towards extra-embryonic lineages^5,6^, compared with their primed counterparts. Therefore, generating and stabilizing nPSCs in vitro is of great importance for a comprehensive modelling of early embryo and foetal anomalies.

Current strategies of primed–naive reversion rely on small-molecule or genetic approaches^7,8^, which are tedious, cost intensive and subject to safety concerns. This warrants the search for robust, scalable, rapid and safe approaches. Structural cues are powerful morphogens in embryo development^9^. At the epiblasts (Epi)–trophectoderm (TE) interface in preimplantation blastocysts, nPSCs show compacted apical cell–cell contacts due to the cellular tension of the surrounding TE^10,11^, which presents an axially symmetric concave geometry^12^ characterized by its height, width and curvature.

Here we hypothesize that a blastocyst-deduced topographical microenvironment on a cell culture substrate can revert cells to naivety by physical means. We anticipate that the cell constraint, mediated by the certain curvature range within the dimensional space of the blastocyst, could elicit reversion. We collectively designate this parameter as the blastocyst-scaled curvature range (BSCR). When the microtopographical units on a substrate encompass the BSCR, they are referred to as BSCR+ motifs. We intend to address the question, whether the reversion requires for BSCR in a radial-symmetric manner, as provided by the Epi–TE interface, or in only a limited number of directions. However, given the impracticality of the side-by-side integration of a multitude of BSCR motifs in a closed three-dimensional architecture like a blastocyst, a structured, that is, open 2.5-dimensional polymeric substrate should be engaged.

To test our hypothesis, the blastocyst motif substrate (BMS) was designed and fabricated featuring thousands of randomly varied motifs, covering not only biological size and shape heterogeneity but also following our reductionist paradigm. Electrical discharge machining was utilized to create the microstructure on metal mould surfaces. The resulting surfaces were then inverted into the targeted BMSs by the injection moulding of polystyrene (Supplementary Table 1 lists the surface parameters). Our hypothesis was validated by employing spatiotemporal analysis, which correlates geometrical information with cellular behaviour. The BSCR—not necessarily radial symmetric—was able to elicit the in situ reversion of primed pluripotent stem cells (PSCs) to a sustainable naive state. The PSCs on BSCR+ motifs presented apical constriction and enhanced E-cad/RAC1 signalling, with weakened cell–substrate interaction. The propagation of physical cues from the BSCR interface to the nuclei through a series of force-dependent elements led to histone modification (H3K27me3 and H3K4me3) at the promoter regions of *Nanog* and *Zic2* and at a distal enhancer region of *Pou5f1*. Topographical cues abstracted from nature’s blueprint offer inherent stability and robustness, which can be easily integrated into the PSC substrate for large-scale implementation in biomanufacturing processes.

## Bioinspired design and characterization of BMS

**Fig. 1:**
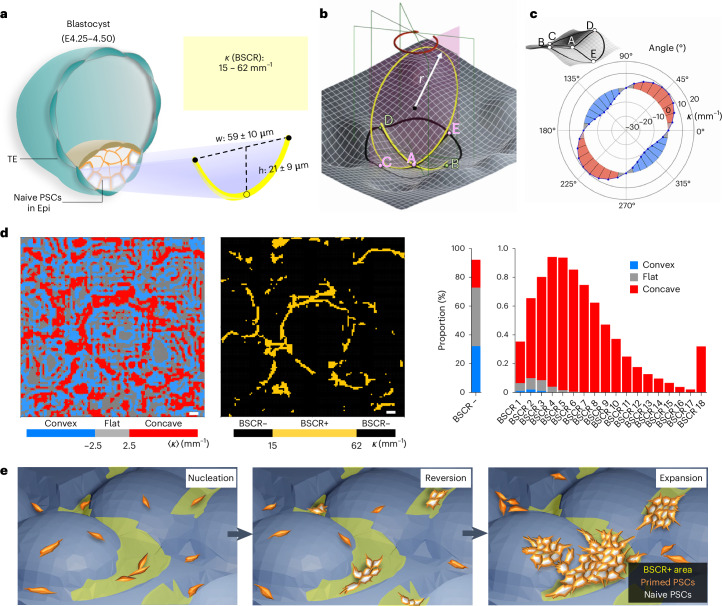
Design and motif analysis of BMS. **a**, Schematic of a mouse blastocyst (preimplantation; E4.25–E4.50). The outer layer of nPSCs experiences curvatures in the BSCR (yellow curve), measured from the Epi–TE interface. **b**–**d**, Computational analysis of BMS surface topographical features. **b**, Scaled three-point approach for evaluating the curvature *κ*, which was analysed in 18 directions for each point using a scale length (CE) of 60 µm, similar to the width of the Epi. **c**, Representative example model showing the curvature value of the given point A in 18 directions. **d**, Left: each point on the topographical surface was allocated a motif according to the mean curvature 〈*κ*〉: convex (〈*κ*〉 ≤ –2.5 mm^–1^, blue), flat (–2.5 mm^–1^ < 〈*κ*〉 < 2.5 mm^–1^, grey), concave (〈*κ*〉 ≥ 2.5 mm^–1^, red). Middle: the BSCR+ area (yellow) included points where *κ* was within the BSCR in at least one direction. Right: proportion of points with different BSCR counts on BMS, correlated with the convex, flat and concave motifs. **e**, Schematic of the overlaid topographical maps of BMS with time-resolved cell images to evaluate the reversion and expansion of PSCs. Source data

We analysed the architecture of blastocysts (embryonic day, E4.25–E4.50)^10^ and determined the dimensional limits of nPSCs in Epi (height, 21 ± 9 µm; width, 59 ± 10 µm), as well as the curvature *κ* of the Epi–TE interface (BSCR), ranging between 15 and 62 mm^−1^ (Fig. 1a and Supplementary Fig. 1).

To grasp both in silico and real-world 2.5-dimensional BMS topographies with respect to the blastocyst curvature properties, we calculated and mapped the spatial information of the BMS surface based on the voxel datasets^13^ from micro-computed tomography (micro-CT) (Supplementary Fig. 2a,b). Noteworthily, we developed a scaled three-point approach (Fig. 1b,c and Supplementary Fig. 3) to measure the asymmetrical curvatures in 18 discrete directions around each surface point on the substrate. A given point was set to a radial distance of 30 µm, that is, half the ~60 µm Epi width, to match the blastocyst scale (Fig. 1a and Supplementary Fig. 1d).

The morphological features of BMS, that is, convex, flat and concave, were then distinguished based on the mean value of the curvatures^13^ 〈*κ*〉 in 18 directions. The individual curvature value *κ* was used to define the BSCR+ area, in which the given point has at least one curvature within the BSCR (Fig. 1d and Supplementary Fig. 2c–e). The representative image of a BMS shows about 8% of the BSCR+ points. The maximum BSCR count lies between 4 and 5. The high proportion of BSCR count at 18 directions indicated the relatively higher proportion of bowl-like structures on the substrates. The BSCR count and morphological features correlate in an intricate way, with the higher number of BSCR located on concave motifs (Fig. 1d).

Establishment of such topographical maps enabled the identification and investigation of specific surface features, including the aforementioned motifs^14^. Correlating these maps with microscopy images showing nPSC distribution (Supplementary Fig. 4), we were able to analyse the in situ effect of geometrical cues on PSC reversion (Fig. 1e).

## BSCR reverts PSCs from primed to naive state

**Fig. 2:**
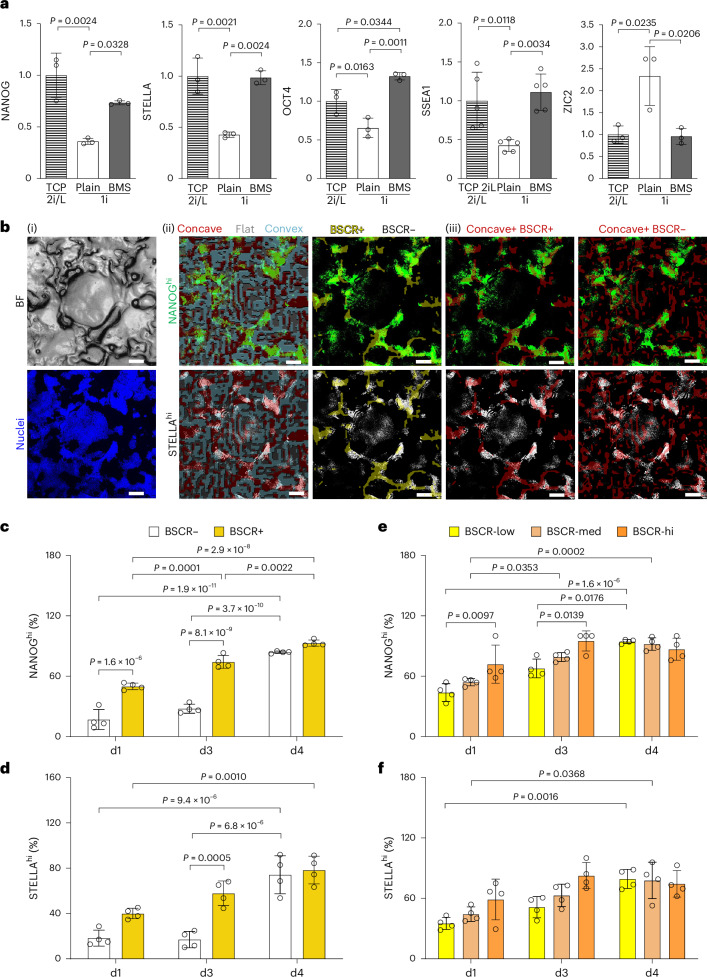
BMS boosts the naivety of PSCs through BSCR motif. **a**, Levels of naive pluripotent markers NANOG, STELLA, OCT4, SSEA1 and primed marker ZIC2 in day-5 PSCs on different substrates. Positive control (TCP, 2i/L) for the naive state was set as 1 (NANOG, STELLA, OCT4 and ZIC2: *n* = 3 biologically independent experiments; SSEA1, *n* = 5 biologically independent experiments). Data are presented as means ± s.d.; statistical significance was calculated via a one-way ANOVA with Bonferroni’s multiple comparisons test. **b**, Representative BMS topographical map and PSC image. Cells were stained on day 3 with Hoechst 33342 to visualize the nuclei of the whole population (blue), whereas the NANOG^hi^ (green) and STELLA^hi^ (white) cells were filtered out to show the naive population. Bright-field (BF) image of BMS and nuclei staining of PSCs (i); naive PSCs overlaid with BMS motif map (ii): concave (red), convex (cyan), flat (grey), BSCR+ (yellow) and BSCR– (black). Naive PSC distribution in BSCR+ and BSCR– areas in the concave region (iii). Scale bars, 100 μm. **c**–**f**, Percentage of nPSCs (NANOG^hi^ and STELLA^hi^) out of the total cells on the indicated motifs was analysed over time to elucidate the effect of BSCR (**c** and **d**) and BSCR counts (**e** and **f**) on PSC naivety. BSCR-low, BSCR-med and BSCR-hi represent BSCR counts of 1–6, 7–12 and 13–18, respectively (*n* = 4 biologically independent experiments; data are presented as means ± s.d.; statistical significance was calculated via a two-way ANOVA with Bonferroni’s multiple comparisons test, for effects of BSCR and time). Source data

**Extended Data Fig. 1:**
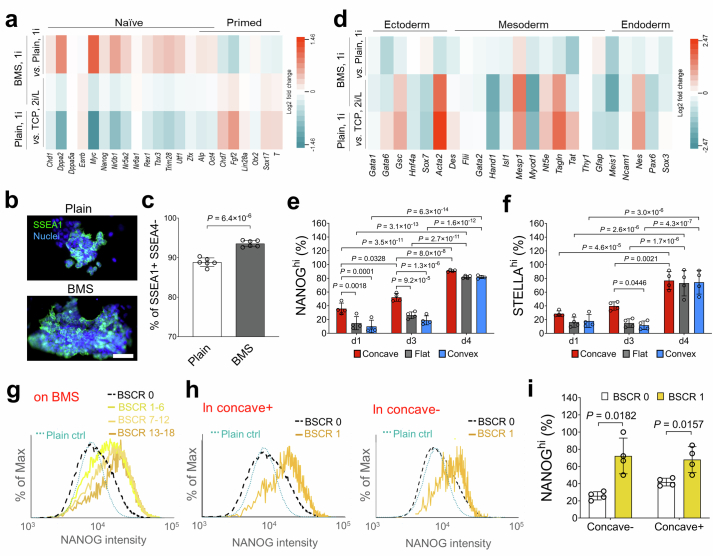
Enhanced naivety of PSCs on BMS. **a**. Real-time PCR array analysis of naive and primed pluripotent gene expression in PSCs on different substrates. **b**. Representative fluorescence microscopic image of SSEA1 (green) and nuclei (blue) of PSCs on Plain and BMS substrate (Images represent the results from 6 independent experiments; Scale bar, 100 μm). **c**. Quantitative analysis of the percentage of SSEA1+/SSEA4– cell population in day-5 PSCs on different substrates (n = 6 biologically independent experiments; Data presented as means ± SD.; Statistical significance was calculated via two-tailed Student’s t test). **d**. Heat map of real-time PCR array screening of germ layer differentiation associated genes in day-5 PSCs. Percentage of NANOG^hi^ (**e**) and STELLA^hi^ (**f**) PSC covered area compared to the whole area of corresponding motifs (n = 4; Statistical significance was calculated via two-way ANOVA with Bonferroni’s multiple comparisons test). **g**. Analysis of intensity of NANOG in cells located in BSCR+ areas with different counts. **h**. Comparison of NANOG intensity of PSCs located in Concave+ and Concave- motifs with one out of 18 directions fitting BSCR (BSCR 1) and without BSCR (BSCR 0). **i**. Quantification of the proportion of NANOG^hi^ cells located at BSCR 0 and BSCR 1 areas in and outside Concave motifs (n = 4 biologically independent experiments; Data presented as means ± SD.; Statistical significance was calculated via two-tailed Student’s t test). Source data

To obtain primed cells, PhiC31 and iPS-MEF-Ng-492B-4 mouse PSCs were chemically adapted for at least three passages to the primed-like state by using 1i medium containing GSK3β inhibitor and AXIN stabilizer (Supplementary Fig. 5), followed by seeding on laminin-coated BMS (Supplementary Fig. 6). Compared with the plain substrate (laminin-coated polystyrene without microstructure), PSCs on BMS exhibited the upregulated naivety genes (Extended Data Fig. 1a), with NANOG, STELLA, OCT4 and SSEA1 proteins at similar levels as the naive control (TCP, 2i/L) (Fig. 2a). For the SSEA4– (a negative marker of mouse PSC) population, the percentage of SSEA1+ (a naive marker of mouse PSC) cells was higher on the BMS (94 ± 1%) than the plain substrate (89 ± 1%) (Extended Data Fig. 1b,c). Contrarily, lineage-specific gene expression was decreased on BMS (Extended Data Fig. 1d). ZIC2, which drives naive–primed transition^15^, was downregulated (Fig. 2a).

To correlate the in situ effect of detailed structural cues on PSC naivety, we sought to discriminate those nPSCs (NANOG^hi^ and STELLA^hi^) using a fluorescence threshold based on the mean fluorescence intensity (MFI) on a plain control (Supplementary Fig. 7) and overlaying those signals with the motif and BSCR maps (Fig. 2b and Supplementary Fig. 4). The number of nPSCs was remarkably higher in concave motifs, than in convex and flat on days 1 and 3 (Fig. 2b and Extended Data Fig. 1e,f). Comparison of concave areas with and without BSCR revealed that BSCR+ motif, rather than concave itself, played the pivotal role to revert the naivety of PSCs (Fig. 2b). These results were supported by analysing NANOG^hi^ and STELLA^hi^ cells on different motifs with varying BSCR counts. On days 1 and 3, a significantly higher percentage of nPSCs was found on BSCR+ compared with BSCR– motif (Fig. 2c,d), and the naivety of nPSCs was correlated with the BSCR counts (Fig. 2e,f and Extended Data Fig. 1g). In particular, even the motifs with BSCR in only one direction (BSCR 1) was sufficient to increase the naivety (Extended Data Fig. 1h,i), indicating that BSCR is the key naive-pluripotency-associated geometrical parameter.

**Fig. 3:**
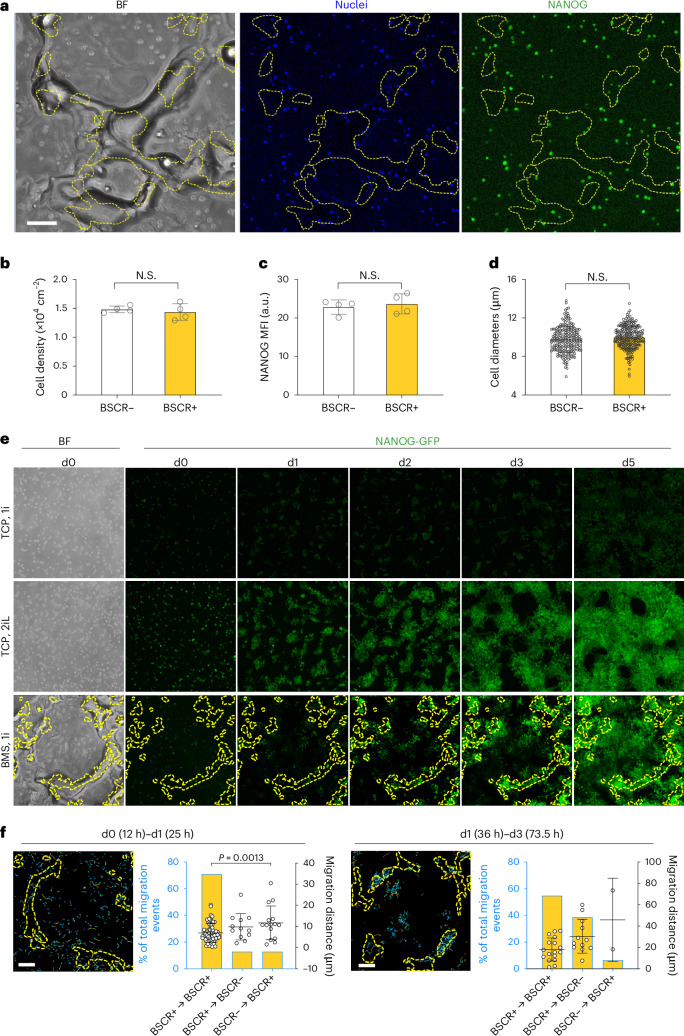
BSCR elicits in situ naivety reversion of PSCs. **a**–**d**, Early-stage cell–BMS interaction was examined 9 h after cell seeding. Representative bright-field, nuclei and NANOG staining images (**a**) and quantitative analysis of cell distribution (**b**), MFI of NANOG (**c**) and size (**d**) of PSCs, located inside and outside the BSCR areas. Scale bars, 100 μm (cell density and NANOG: *n* = 4; cell size: *n*_cell_ = 220 and 228 for the BSCR– and BSCR+ groups, respectively; data are presented as means ± s.d.; statistical significance was calculated via a two-tailed Student’s *t*-test; N.S., non-significance). **e**, Dynamics of the NANOG-GFP expression of PSCs. Cells cultured on TCP with 1i and 2i/L media were used as negative and positive controls of naivety, respectively. Images represent the results from three independent experiments. Scale bar, 100 μm. **f**, Migration of PSCs (left: whole population; right: NANOG-GFP^hi^ nPSCs) inside the BSCR+ area and crossing over the BSCR border on BMS in the indicated time frames. Images were recorded within 60 min (left) and 90 min (right) intervals. Scale bars, 100 μm (*n*_cell_ = 93 and *n*_cell_ = 31 for d0–d1 and d1–d3 cell tracking; data are presented as means ± s.d.; statistical significance was calculated via a one-way ANOVA with Bonferroni’s multiple comparisons test). The outlines of the BSCR+ areas were illustrated with dashed yellow lines. Source data

**Extended Data Fig. 2:**
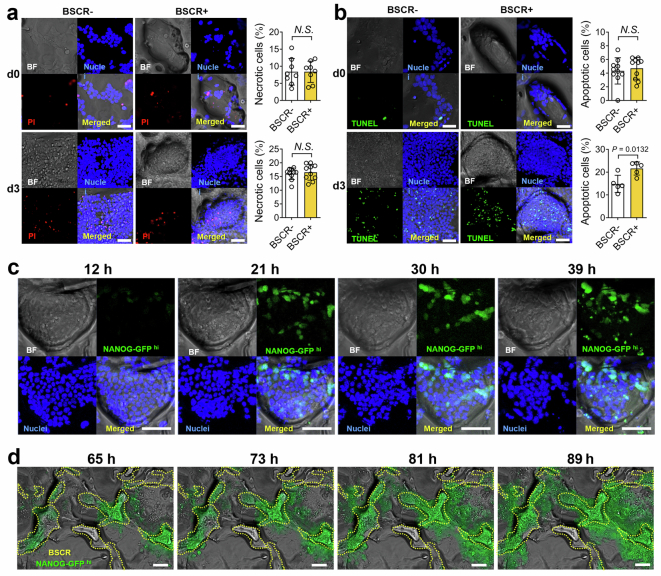
Primed-to-naive reversion of PSCs on BMS. Representative images and quantitative analysis of PSC necrosis (**a**) and apoptosis (**b**) in BSCR+ and BSCR- areas at day 0 (9 h post cell seeding; n = 8 images for necrosis and n = 10 images for apoptosis) and day 3 (n = 10 images for necrosis and n = 5 images for apoptosis; Data presented as means ± SD.; Statistical significance was calculated via two-tailed Student’s t test; *N.S*. statistically non-significance). **c**. Detection of NANOG-GFP^hi^ nPSCs on BSCR+ area of BMS from 12 h to 39 h post cell seeding. **d**. Expansion of NANOG-GFP^hi^ nPSCs from BSCR+ to BSCR- areas. The outlines of BSCR+ areas were illustrated with yellow dash lines. Images in **a-d** represent the results from 3 independent experiments. Scale bar, 100 μm. Source data

To validate the in situ primed–naive reversion, rather than other mechanisms such as cell selection and migration, we investigated the cell–substrate interaction from the initial cell attachment. After seeding, the cells exhibited a random distribution on different motifs of the BMS, with similar density, NANOG expression and cell size 9 h post-cell seeding (Fig. 3a–d). The BSCR motif did not affect the necrosis of PSCs. The apoptosis level of PSCs in the BSCR+ area remained similar to BSCR– on day 0, but increased to a significantly higher level on day 3 (Extended Data Fig. 2a,b). This might be attributed to the higher sensitivity to cellular stress in naive PSCs than their primed counterparts^16,17^, as a result of elevated mitochondrial priming^18^. In addition, the high cell confluence in the BSCR+ area, resulting from the enhanced proliferation of nPSCs, could trigger apoptotic cell death^19,20^.

**Extended Data Fig. 3:**
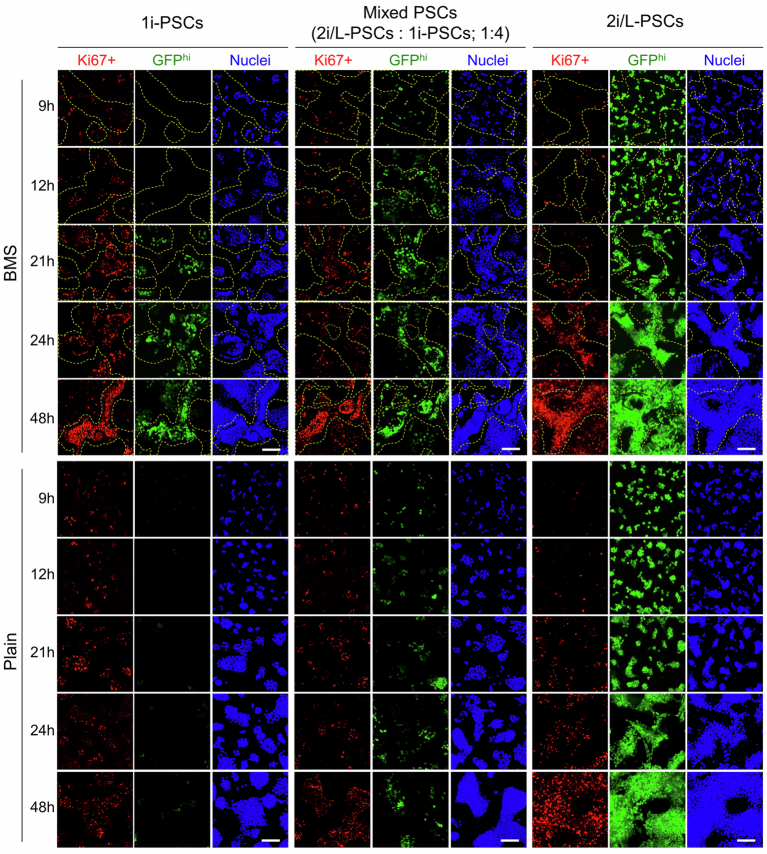
PSC naivety and proliferation activity on different substrates. Representative fluorescence images of Ki67+ proliferating cells, NANOG-GFP^hi^ naive PSCs in BSCR+ and BSCR- areas of BMS, and on Plain substrate (Images represent the results from 3 independent experiments. Scale bar, 100 μm). The outlines of BSCR+ areas were illustrated with yellow dash lines. 1i-PSCs, Mixed PSCs (Naive:Primed = 1:4 in number) were maintained under primed PSC culture condition in 1i medium. 2i/L-PSCs used as positive control were cultured under naive PSC culture condition in 2i/L medium.

**Extended Data Fig. 4:**
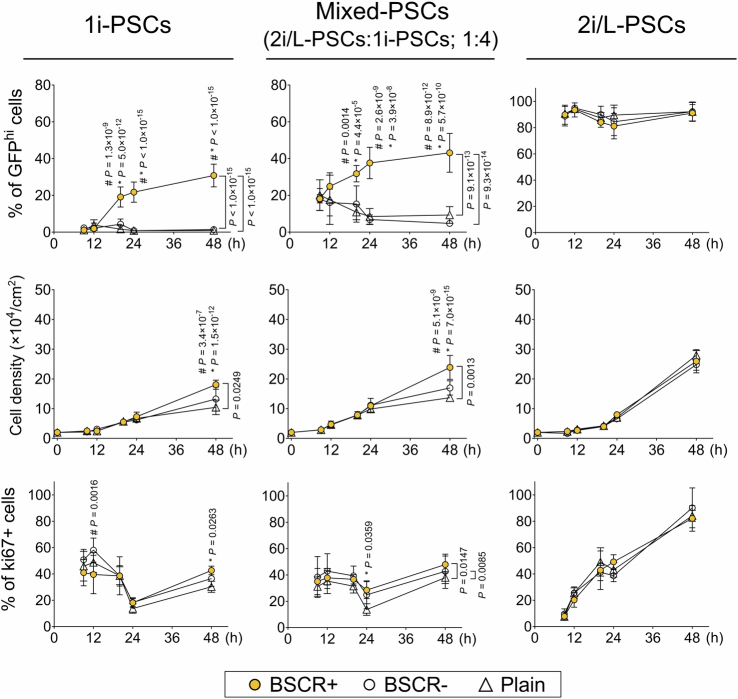
Quantification of PSC naivety and proliferation activity on different substrates. The NANOG-GFP^hi^ proportion, cell density and percentage of Ki67+ proliferating cells in BSCR + , BSCR- areas of BMS and on Plain substrate within 48 h post cell seeding were quantified based on the fluorescence images from 5 biologically independent experiments (For GFP^hi^ cell analysis over time, 1i-PSCs group: n_BSCR+_=35, n_BSCR-_=26, n_Plain_ = 25; Mixed PSCs group: n_BSCR+_=27, n_BSCR-_=26, n_Plain_ = 26; 2i/L-PSCs group: n = 25. For cell density analysis, 1i-PSCs group: n_BSCR+_=44, n_BSCR-_=32, n_Plain_ = 30; Mixed PSCs group: n_BSCR+_=33, n_BSCR-_=33, n_Plain_ = 32; 2i/L-PSCs group: n = 30. For Ki67+ cell analysis, 1i-PSCs group: n_BSCR+_=35, n_BSCR-_=26, n_Plain_ = 25; Mixed PSCs group: n_BSCR+_=27, n_BSCR-_=27, n_Plain_ = 26; 2i/L-PSCs group: n = 25. Data presented as means ± SD.; Statistical significance was calculated via two-way ANOVA with Bonferroni’s multiple comparisons test, for effect of BSCR; *BSCR+ *vs*. Plain, #BSCR+ *vs*. BSCR-). Source data

To evaluate the reversion kinetics, we utilized time-lapse imaging to track live cells using a PSC line with NANOG reporter–GFP^21^. The gradually increased GFP signals was observed in the BSCR+ area as early as 21 h after cell seeding (Fig. 3e and Extended Data Fig. 2c). By analysing the migration of single cells, we further confirmed the in situ reversion within the BSCR+ motif. From day 0 to day 1, the migration distance of the cells on BMS was limited to 10 µm. From day 1 to day 3, rare migration was observed from BSCR– to BSCR+ (Fig. 3f). These results excluded the possibility that nPSCs in BSCR+ are from BSCR– as a result of remote migration. In particular, more nPSCs were found in the BSCR– area at later time points (Fig. 3e), which is consistent with the results that a similar percentage of nPSCs were found in different motifs on day 4 (Fig. 2c,d and Extended Data Fig. 1e,f). The analysis of the trajectory video revealed that nPSCs in the BSCR– area are mainly from the BSCR+ area, with high confluence and subsequent expansion/migration (Fig. 3f, Extended Data Fig. 2d and Supplementary Videos 1 and 2). The impact of motifs on PSC naivety reversion and proliferation was further examined with time-resolved imaging analysis (Extended Data Figs. 3 and 4). Consistent with the reported results^22–24^, the naive cells (2i/L-PSCs) proliferated faster than the primed counterparts (1i-PSCs) and the naive/primed mixed PSCs, showing the highest increasing rate of cell density on all the tested motifs. BSCR+ motif could revert primed cells to a more naive state. For the 1i-PSCs and mixed PSCs, which contains the primed cells, a significant increase in GFP^hi^ cell percentage was observed 21 h after cell seeding in the BSCR+ area, compared with the BSCR– and plain substrates. However, the percentage of Ki67+ cells remained at a similar level at/before these time points. This result indicated that the increased number of naive cells in the BSCR+ area at the early stage is mainly attributed to the reversion of—rather than the proliferation of—naive cells.

**Extended Data Fig. 5:**
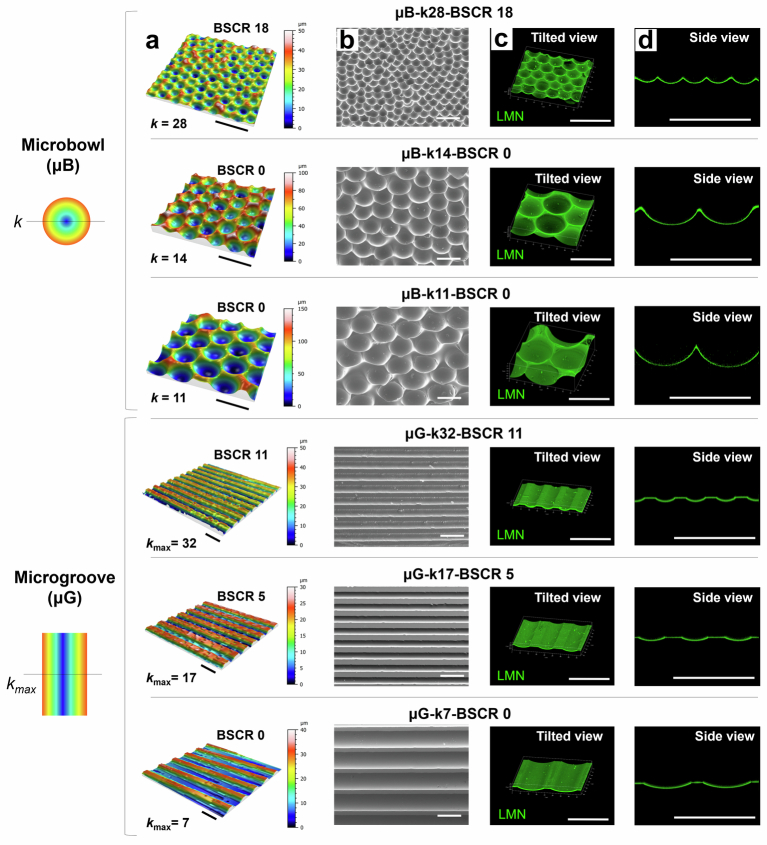
Topography of microbowl and microgroove substrates. **a**. Height map reconstruction of substrates based on optical profilometry analysis. **b**. Representative scanning electron microscopic images of microstructured substrates. Confocal microscopic images of tilted-view (**c**) and side-view (**d**) of laminin-coated microbowls and microgrooves (Images represent the results from 3 independent experiments; Scale bar, 200 μm).

**Extended Data Fig. 6:**
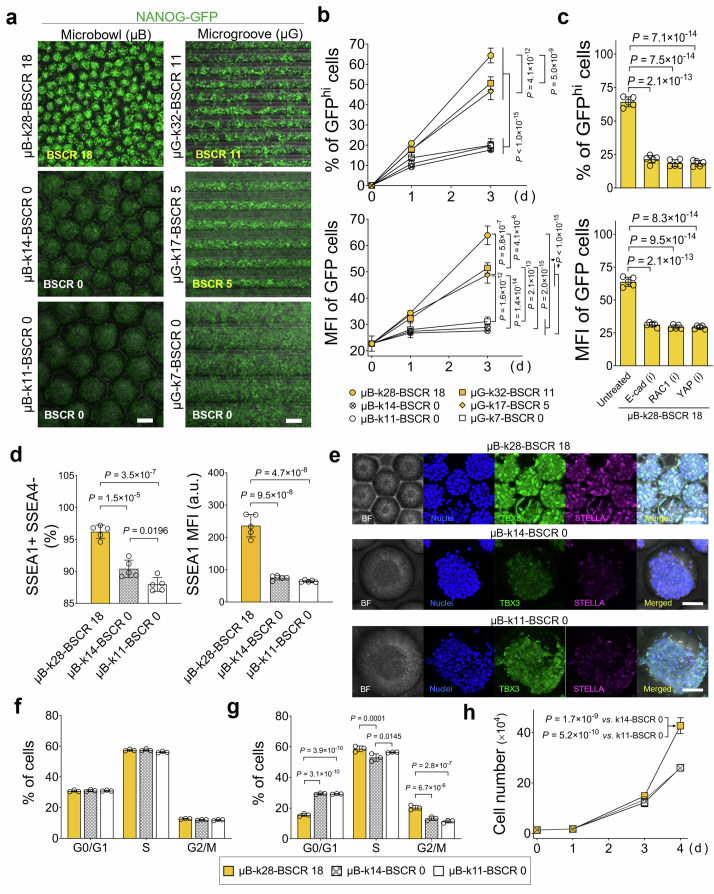
Primed-to-naive reversion and expansion of PSCs on substrates with uniform motifs. **a**. Representative confocal microscopic images of NANOG-GFP PSCs on microbowls and microgrooves with different BSCR counts at day 3 after cell seeding (Fluorescence images represent the results from 3 independent experiments; Scale bar, 100 μm). **b**. Quantification of the area fraction of GFP^hi^ in GFP+ cells and MFI of GFP in PSCs growing on microbowls and microgrooves with different BSCR counts for 3 days (n = 5 biologically independent experiments; Data presented as means ± SD.; Statistical significance was calculated via two-way ANOVA with Bonferroni’s multiple comparisons test, for effects of BSCR and time). **c**. the area fraction of GFP^hi^ in GFP+ cells and MFI of GFP in day-3 PSCs on BSCR+ microbowls with and without E-cad, RAC1 and YAP inhibition (n = 5 biologically independent experiments; Data presented as means ± SD.; Statistical significance was calculated via one-way ANOVA with Bonferroni’s multiple comparisons). **d**. Quantification of fraction and MFI of SSEA1+ PSCs on BSCR+ and BSCR- microbowls (n = 5 biologically independent experiments; Data presented as means ± SD.; Statistical significance was calculated via one-way ANOVA with Bonferroni’s multiple comparisons). **e**. Representative confocal images showing the alteration of TBX3 and STELLA in PSCs on microbowls with different BSCR counts (Images represent the results from 3 independent experiments; Scale bar, 50 μm). Cell cycle progression patterns of day-1 (**f**) and day-3 (**g**) PSCs on microbowls with different BSCR counts (n = 3 biologically independent experiments; Data presented as means ± SD.; Statistical significance was calculated via two-way ANOVA with Bonferroni’s multiple comparisons test). **h**. Expansion curves of PSCs on microbowls with different BSCR counts over time (n = 3 biologically independent experiments; Data presented as means ± SD.; Statistical significance was calculated via two-way ANOVA with Bonferroni’s multiple comparisons test, for effects of BSCR and time). Source data

Next, to verify the effect of BSCR on naivety reversion, we created substrates with uniform microbowls (radial symmetry) and microgrooves (axial symmetry) (Extended Data Fig. 5 and Supplementary Tables 2 and 3). Consistent with BMS, reversion correlates with the BSCR counts, regardless of the microstructure shape. The microbowls without BSCR, despite their similarity in shape with a blastocyst, demonstrated a lower reversion capacity than the microgrooves with BSCR (Extended Data Fig. 6a,b). The uniform microstructures with higher BSCR counts induced higher levels of naivety (NANOG-GFP, SSEA1, TBX3 and STELLA) (Extended Data Fig. 6). When PSCs were cultured on substrates with microbowls with and without BSCR, they showed similar proliferation profile from days 1 to 3 (Extended Data Fig. 6f–h), but the higher percentage of GFP^hi^ PSCs could be found as early as day 1 on microbowl-containing BSCR (Extended Data Fig. 6b). This observation is in line with that on BMS, suggesting that the increase in naive cells in the BSCR+ area in the early stage is not from proliferation but is a result of naivety reversion.

## BSCR triggers mechanosensation and histone modification

**Extended Data Fig. 7:**
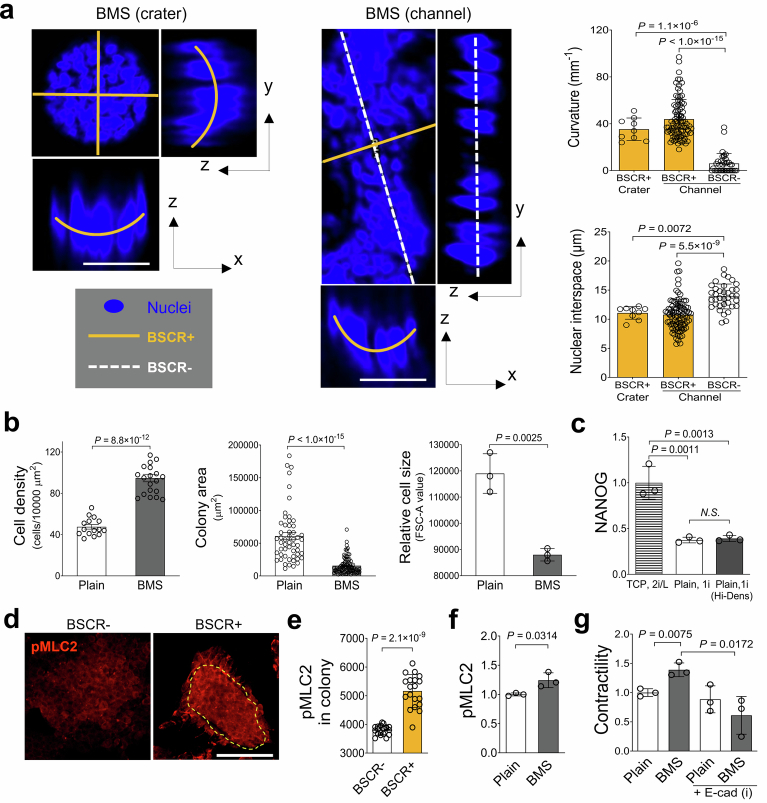
PSC compaction on BMS. **a**. Representative top- and side-view of confocal microscopic images and quantitative analysis showing spatial organization of PSC colonies in crater and channel like areas on BMS. Cell nuclei were stained with DAPI (Data acquired from 9 biologically independent experiments; BSCR+ Crater: n = 9, BSCR+ Channel: n = 90, BSCR- Channel: n = 36; Data presented as means ± SD.; Statistical significance was calculated via one-way ANOVA with Bonferroni’s multiple comparisons test). **b**. Imaging analysis of cell density, top-view colony area, and flow cytometry analysis of relative cell size (Cell density: ncolony=15 and 20 for Plain and BMS substrate from 10 biologically independent samples; Colony area: n = 8 and 22, ncolony=53 and 91 for Plain and BMS substrate from 10 biologically independent samples, respectively; Cell size: n = 3 biologically independent samples; Data presented as means ± SD.; Statistical significance was calculated via two-tailed Student’s t test; *N.S*., non-significance). **c**. The flow cytometry analysis of NANOG of day-5 PSCs on Plain substrates with and without high cell seeding density (5×10^4^ /cm^2^). TCP, 2i/L control was set as 1 (n = 3 biologically independent experiments; Data presented as means ± SD.; Statistical significance was calculated via one-way ANOVA with Bonferroni’s multiple comparisons test; *N.S*., non-significance). Representative confocal images and (**d**) the quantification of pMLC2 level (**e**) in PSC colonies at BSCR+ and BSCR- areas (Scale bar, 100 μm; n_colony_ = 19 from 3 biologically independent experiments; Data presented as means ± SD.; Statistical significance was calculated via two-tailed Student’s t test). **f**. Comparison of pMLC2 levels of cells using flow cytometry (n = 3 biologically independent experiments; Data presented as means ± SD.; Statistical significance was calculated via two-tailed Student’s t test). **g**. Contractility assay based on the PSCs/collagen gel size measurement. The cells were harvested from Plain and BMS with/without E-cad inhibition (n = 3 biologically independent experiments; Data presented as means ± SD.; Statistical significance was calculated via two-tailed Student’s t test). Source data

PSCs inside the BSCR+ area presented the morphological characteristics of naive cells, with compacted colony and decreased cell interspace along the direction of BSCR (Supplementary Figs. 1f and 7a,b). The mere increase in the cell density on a plain substrate did not increase the NANOG level (Extended Data Fig. 7c), suggesting the indispensable role of BSCR curvature on naivety reversion.

**Fig. 4:**
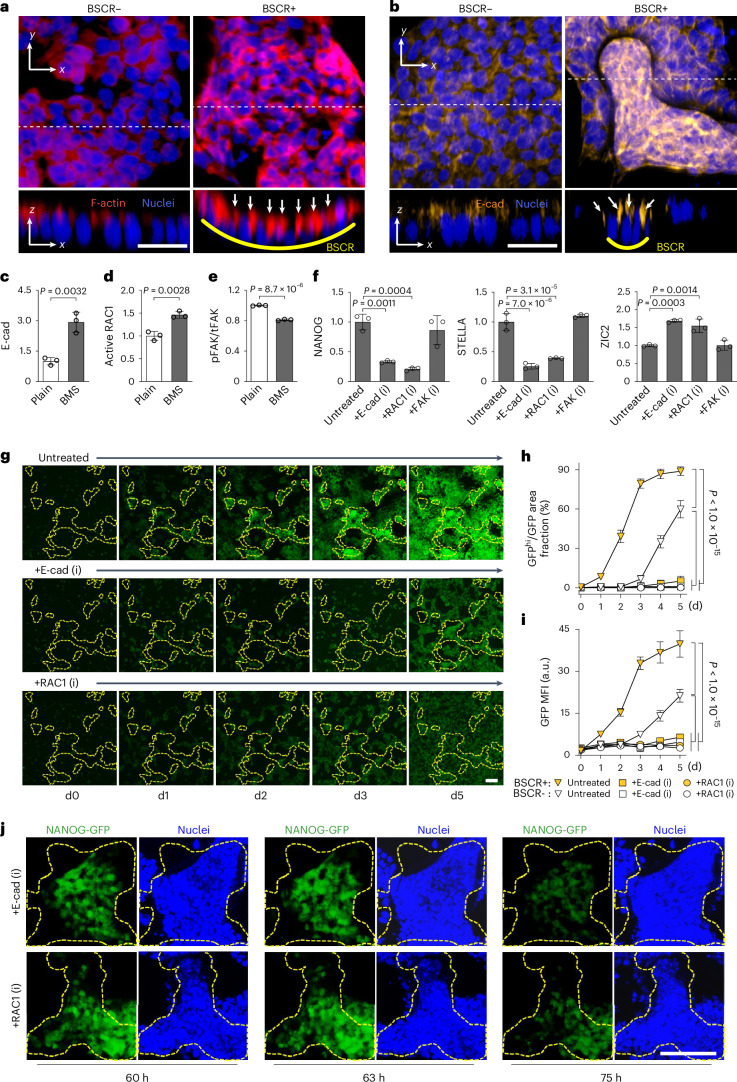
BMS reverts PSCs to naive state via E-cad/RAC1 signalling. **a**,**b**, Representative immunofluorescence images of F-actin (red) (**a**) and E-cad (orange) (**b**) in PSC colonies on BMS: top view (*x*–*y* plane) and side view (*x*–*z* plane; optical cross section from the top view along the dotted white lines) (cell nuclei were stained with DAPI; images represent the results from three independent experiments). Scale bar, 50 µm. **c**–**e**, Quantification of E-cad expression using flow cytometry (**c**), active RAC1 (**d**) and FAK (**e**) activity (Y397-phosphorylated FAK (pFAK)/total FAK (tFAK) ratio) with ELISA, for PSCs on plain substrate and BMS (*n* = 3 biologically independent experiments; data are presented as means ± s.d.; statistical significance was calculated via a two-tailed Student’s *t*-test). **f**, Level of NANOG, STELLA and ZIC2 in day-5 PSCs on BMSs with and without E-cad, RAC1 and FAK inhibition. The value of untreated PSCs was set as 1 (*n* = 3 biologically independent experiments; data are presented as means ± s.d.; statistical significance was calculated via a one-way ANOVA with Bonferroni’s multiple comparisons). **g**, Dynamic changes in NANOG-GFP in PSCs growing on BMS for 5 days with and without E-cad and RAC1 inhibition. Scale bar, 100 μm. **h**, Fraction of nPSCs (GFP^hi^) out of the total GFP+ cells on different motifs over time, with and without inhibitor treatment. The result was expressed as a ratio of areas covered by GFP^hi^ cells and GFP+ cells (*n* = 3 biologically independent experiments; data are presented as means ± s.d.; statistical significance was calculated via a two-way ANOVA with Bonferroni’s multiple comparisons test, for effects of BSCR and time). **I**, MFI of GFP in the BSCR+ and BSCR– areas over time (*n* = 3 biologically independent experiments; data are presented as means ± s.d.; statistical significance was calculated via a two-way ANOVA with Bonferroni’s multiple comparisons test, for effects of BSCR and time). **j**, Representative time-lapse images showing the inhibition of E-cad/RAC1 signalling abolished the naivety of PSCs inside the BSCR+ area. Images represent the results from three independent experiments. Scale bar, 100 μm. For **g** and **j**, the BSCR+ areas are circled with dashed yellow lines. Source data

It is known that the preservation of PSC naivety can be strengthened by cytoskeleton-bound cell–cell junctions^25–27^. Here cell clusters in the BSCR+ area displayed strong apical F-actin and E-cad compared with the BSCR– area (Fig. 4a,b). At the population level, E-cad and its stabilizer RAC1 (refs. ^28–30^) were upregulated on BMS (Fig. 4c,d). The inhibition of E-cad and RAC1 significantly decreased BSCR-mediated naivety reversion on both BMS (Fig. 4f–i) and substrates with uniformed microbowls (Extended Data Fig. 6c). Furthermore, the nPSCs rapidly lost their naivety on E-cad and RAC1 inhibition, suggesting the essential role of E-cad/RAC1 signalling in the preservation of naivety (Fig. 4j). We further investigated the intracellular components related to mechanical force propagation. A strong upregulation of pMLC2 and a higher cell contractility were observed in BMS–PSCs, which was abolished on E-cad neutralization (Extended Data Fig. 7d–g), suggesting the intermediary role of cell–cell adhesion in force transmission. PSCs on BMS exhibited a weakened cell–substrate interaction, with about 20% decrease in active FAK (phosphorylated/total FAK ratio) (Fig. 4e), which is in agreement with the previous report that concave curvature weakened the cell–substrate interaction^31^. The naive markers NANOG, STELLA and primed marker ZIC2 in BMS–PSCs were not altered following FAK inhibition (Fig. 4f). These results suggested that the naivety of BMS–PSCs indispensably relied on cell–cell adhesion and E-cad/RAC1 signalling, but not on FAK-mediated cell–substrate adhesion.

**Extended Data Fig. 8:**
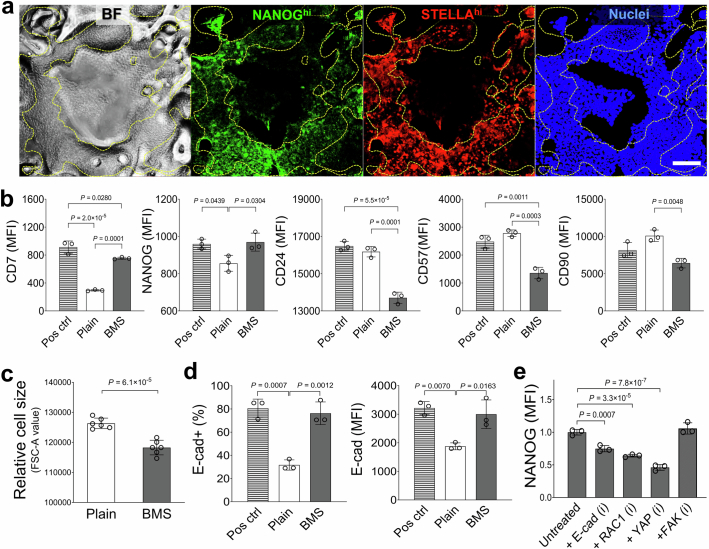
Primed-to-naive reversion of human PSCs on BMS. **a**. Representative bright field (BF) image and laser scanning microscopic images of human PSCs on BMS at day 5. The nuclei (blue) were stained with Hoechst 33342 to visualize the whole population, and the NANOG^hi^ (green) and STELLA^hi^ (red) cells were filtered out to show the naive population (Images represent the results from 3 independent experiments; Scale bars, 100 μm). **b**. Flow cytometry analysis of naive markers (CD7, NANOG) and primed markers (CD24, CD57, CD90) of day-8 human PSCs adapted in RSeT medium on TCP (Pos ctrl), on Plain and BMS substrates in IPS-brew medium (n = 3 biologically independent experiments; Data presented as means ± SD.; Statistical significance was calculated via one-way ANOVA with Bonferroni’s multiple comparisons test). **c**. Flow cytometry analysis of relative cell size (based on FSC-A value) of human PSCs cultured on Plain and BMS surfaces (n = 6 biologically independent experiments; Data presented as means ± SD.; Statistical significance was calculated via two-tailed Student’s t test). **d**. Quantitative analysis of E-cad+ fractions and E-cad expression levels of cells under different culture conditions (n = 3 biologically independent experiments; Data presented as means ± SD.; Statistical significance was calculated via one-way ANOVA with Bonferroni’s multiple comparisons test). **e**. The level of NANOG in day-8 human PSCs on BMS with and without E-cad, RAC1 and FAK inhibition. The level of untreated PSCs was set as 1 (n = 3 biologically independent experiments; Data presented as means ± SD.; Statistical significance was calculated via one-way ANOVA with Bonferroni’s multiple comparisons). Source data

These findings led us to further examine the reversion of human PSCs, which might require different intracellular signalling compared with mouse PSCs^2,3^. Similarly, BSCR+ motifs efficiently reverted human PSCs to a naive state (Extended Data Fig. 8a), with enhanced naive markers CD7 and NANOG and reduced prime markers CD24, CD57 and CD90 (refs. ^32–34^) (Extended Data Fig. 8b). Cells were more compacted on BMS than on the plain substrate, showing smaller cell size (Extended Data Fig. 8c) and strongly activated E-cad (Extended Data Fig. 8d). The level of NANOG was significantly reduced on the inhibition of E-cad, RAC1 and YAP, but not affected by FAK inhibition (Extended Data Fig. 8e). These results might indicate the similarity in key components for curvature sensing and naivety reversion between mouse and human PSCs, highlighting the feasibility of BMS for applications in human PSCs.

**Fig. 5:**
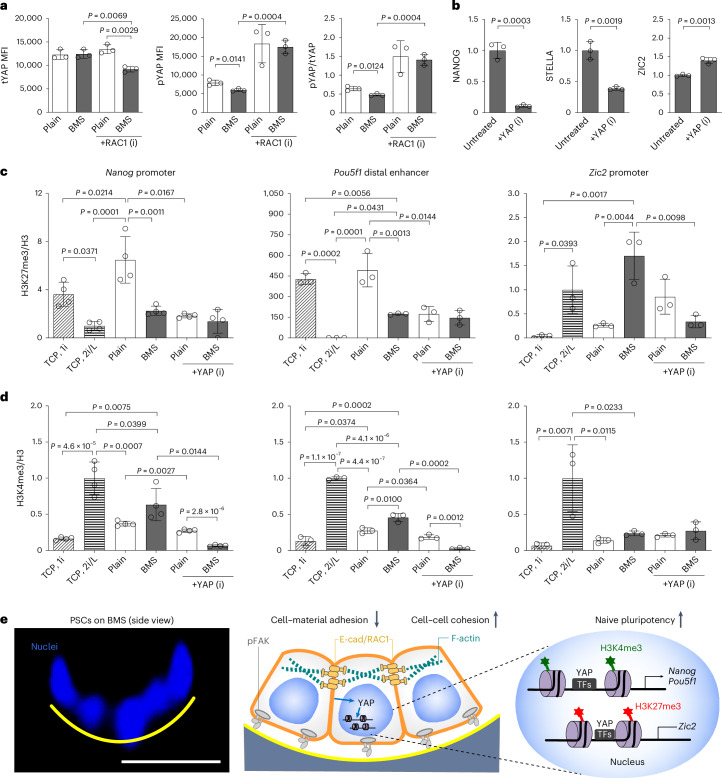
Epigenetic reversion of PSCs to the naive state on BMS mediated by YAP and histone modification. **a**, Flow cytometry analysis of total YAP (tYAP), S127-phosphorylated YAP (pYAP) and pYAP/tYAP ratio in PSCs from plain substrate and BMS, with and without RAC1 inhibition (*n* = 3 biologically independent experiments; data are presented as means ± s.d.; statistical significance was calculated via a two-tailed Student’s *t*-test for comparing plain versus BMS–PSCs or untreated versus inhibition groups). **b**, Level of NANOG, STELLA and ZIC2 in day-5 PSCs on BMS with and without YAP inhibition. The level of untreated group was set as 1 (*n* = 3 biologically independent experiments; data are presented as means ± s.d.; statistical significance was calculated via a two-tailed Student’s *t*-test). **c**,**d**, ChIP PCR analysis of H3K27me3 (**c**) and H3K4me3 (**d**) levels at the promoter regions of *Nanog* and *Zic2* and at distal enhancer region of *Pou5f1* in the absence and presence of YAP inhibitor. Data were normalized by the total H3 value in each group. Positive control (TCP, 2iL) group was set as 1 (*Nanog* promoter: *n* = 4 biologically independent experiments; *Pou5f1* distal enhancer and *Zic2* promoter: *n* = 3 biologically independent experiments; data are presented as means ± s.d.; statistical significance was calculated via a one-way ANOVA with Bonferroni’s multiple comparisons test for groups without YAP inhibition and via a two-tailed Student’s *t*-test for comparing inhibition and the corresponding untreated groups). **e**, Proposed mechanism for nativity reversion at the epigenetic level. Left: side view of the mouse PSC nuclei in the BSCR+ area (yellow curve). Right: scheme of intracellular and intranuclear signalling events triggered by BSCR. TF, transcription factor. Source data

**Extended Data Fig. 9:**
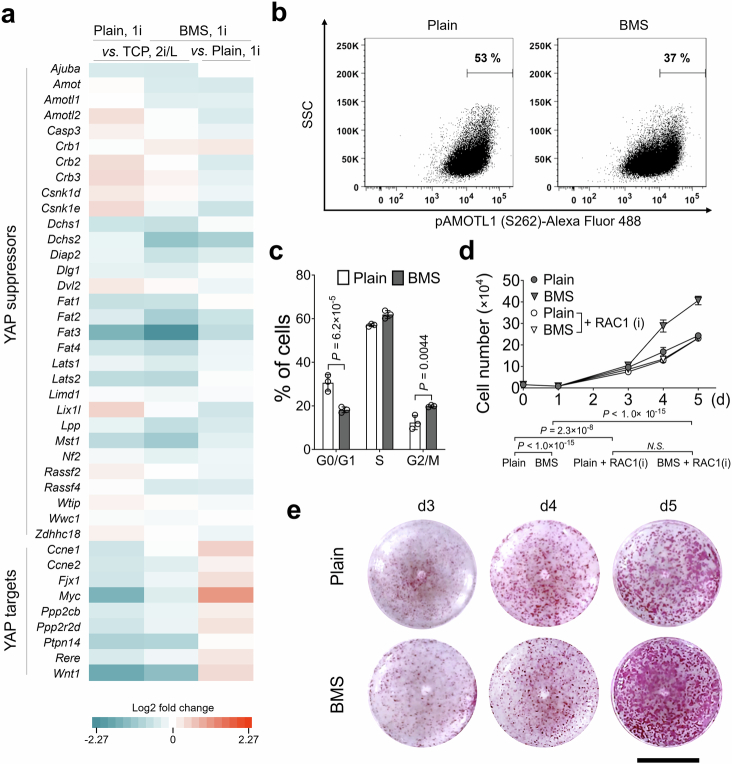
YAP activation and cell proliferation in response to culture on a BMS substrate. **a**. Real-time PCR array analysis of YAP signaling suppressor and target genes in day-3 PSCs from Plain and BMS substrates. **b**. The representative flow cytometry dot plots showing the phosphorylated level of AmotL1 (S262) in PSCs in the indicated conditions. **c**. Percentage of cells in the phases of cell cycle on Plain and BMS substrates (n = 3 biologically independent experiments; Data presented as means ± SD.; Statistical significance was calculated via two-way ANOVA with Bonferroni’s multiple comparisons test). **d**. Growth curve of PSCs on Plain and BMS substrates, with and without RAC1 inhibition (n = 9 biologically independent experiments; Data presented as means ± SD.; Statistical significance was calculated via two-way ANOVA with Bonferroni’s multiple comparisons test; *N.S*, non-significance). **e**. Alkaline phosphatase staining of PSCs on Plain and BMS at indicated time points. Image represents the results from 3 biologically independent experiments. Scale bar, 5 mm. Source data

E-cad/RAC1 signalling can further mediate the activation of YAP^35–37^, which regulates stem cell fate and pluripotency as a mechanotransducer^38–40^. Compared with the plain group, the total YAP level was unaltered, whereas inactive phosphorylated YAP was significantly reduced in BMS–PSCs (Fig. 5a). PCR array analysis showed that YAP downstream target genes were upregulated, whereas upstream suppressor genes were downregulated on BMS (Extended Data Fig. 9a). In particular, phosphorylated AmotL1—an inhibitor of YAP—decreased in BMS–PSCs (Extended Data Fig. 9b). Given the central role of YAP in cell cycle progression^40,41^, we analysed the cell cycles and found a naivety-featured rapid G1/S transition, a higher proliferation rate and clonogenic potential in BMS–PSCs (Extended Data Fig. 9c–e). The inhibition of RAC1 reduced the YAP activity on BMS (Fig. 5a) and resulted in decreased cell proliferation (Extended Data Fig. 9d), suggesting the involvement of E-cad/RAC1 signalling in YAP activation. The role of YAP on PSC naivety was further validated via the inhibition of YAP, which significantly decreased NANOG and STELLA but upregulated ZIC2 (Fig. 5b and Extended Data Fig. 6c).

Further, we performed chromatin immunoprecipitation (ChIP) polymerase chain reaction (PCR) to gain a better understanding of YAP in the epigenetic regulation of pluripotency genes^40,42^. BMS–PSCs showed significantly decreased H3K27me3 but increased H3K4me3 occupancy on ‘naive loci’ including *Nanog* promoter and *Pou5f1* distal enhancer. There were increased H3K27me3 on the promoter region of *Zic2* in BMS–PSCs (Fig. 5c). YAP inhibition significantly decreased the level of H3K4me3 on *Nanog* promoter and *Pou5f1* distal enhancer, as well as the level of H3K27me3 on *Zic2* promoter (Fig. 5c,d), suggesting that YAP activation in BMS–PSCs played a key role in the histone modification of pluripotency genes. These data suggest a mechanistic relationship between BSCR, enhanced E-cad/RAC1 signalling and YAP activation, which consequently increased permissive versus repressive histone patterns in line with a gain of naivety (Fig. 5e).

## BSCR stabilizes PSC naivety

**Fig. 6:**
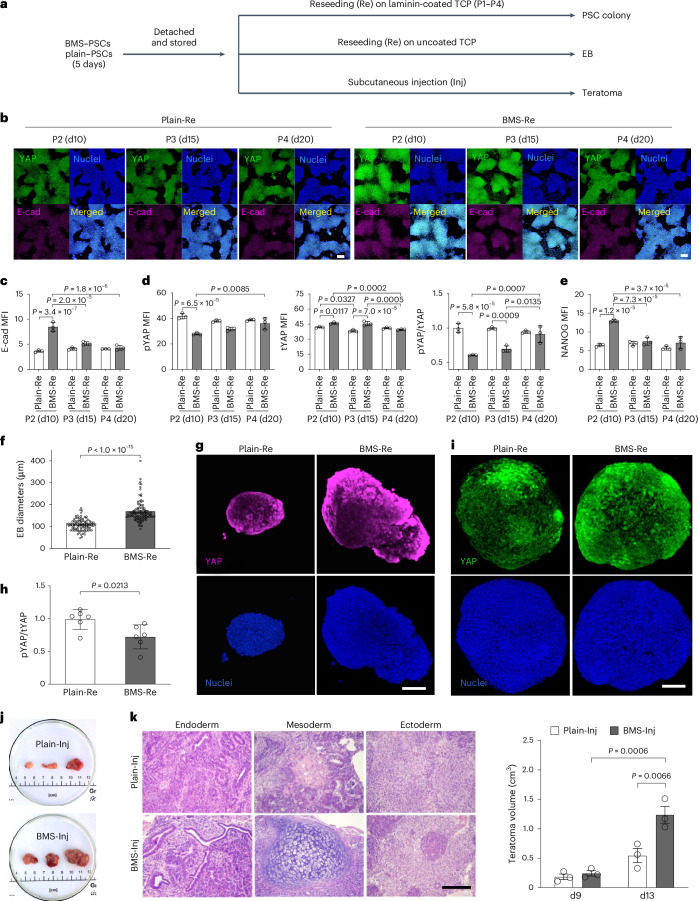
BMS stabilizes naive pluripotency of PSCs. **a**, Scheme for analysing the naive pluripotency stability of PSCs preconditioned from substrates. PSCs were preconditioned on plain substrate and BMS for 5 days, followed by reseeding on laminin-coated TCP for monolayer culture, on uncoated TCP for EB formation and by injection for teratoma generation. **b**, Representative images showing the dynamic change in YAP and E-cad levels in PSCs reseeded on laminin-coated TCP. **c**–**e**, Quantitative analysis of E-cad levels (**c**), pYAP levels, tYAP levels, pYAP/tYAP ratio (**d**) and NANOG levels (**e**) in plain-Re and BMS-Re PSCs (*n* = 3 biologically independent experiments; data are presented as means ± s.d.; statistical significance was calculated via a two-way ANOVA with Bonferroni’s multiple comparisons). **f**–**h**, Size (**f**), YAP staining (**g**) and YAP phosphorylation level (**h**) of EBs, which were formed from reseeded PSCs in uncoated 96-well flat TCPs. EBs grew for 2 days (**f**) and 3 days (**g** and **h**). (**f**: *n* = 100 EBs from three biologically independent experiments; data are presented as means ± standard error of the mean; statistical significance was calculated via a two-tailed Student’s *t*-test; **g**: scale bar, 100 µm; **h**: *n* = 6 biologically independent experiments; data are presented as means ± s.d.; statistical significance was calculated via a two-tailed Student’s *t*-test; the value of the plain-Re group was set as 1). **i**, YAP staining of EBs, which were formed by PSCs derived from BMS and plain substrates in a V-bottom culture plate for 5 days to achieve a similar EB diameter. Scale bar, 100 μm. **j**, Representative images of teratomas formed in mice by the injection of preconditioned PSCs. **k**, Representative histological images and volume of teratomas derived from injected PSCs. Scale bar, 250 μm. *n* = 3 biologically independent experiments; data are presented as means ± standard error of the mean; statistical significance was calculated via a two-way ANOVA with Bonferroni’s multiple comparisons test. Source data

**Extended Data Fig. 10:**
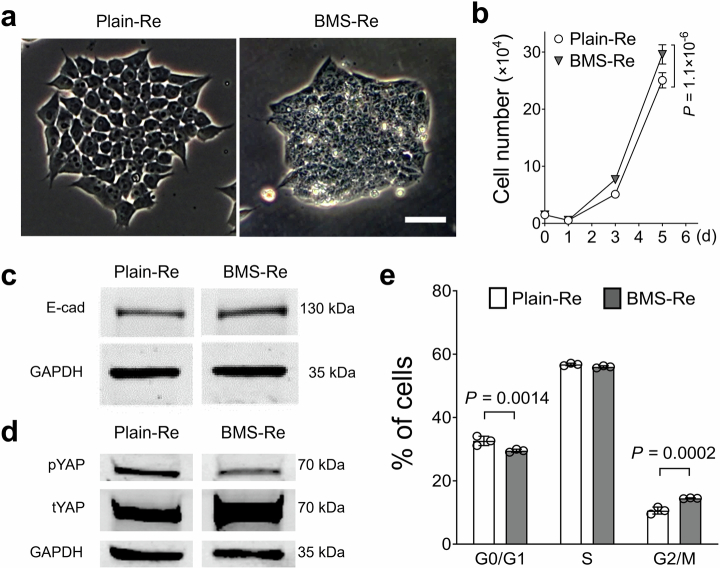
BMS stabilizes naive pluripotency of PSCs. **a**. Morphology of reseeded PSCs pre-conditioned on different substrates. Scale bar, 50 μm. **b**. Proliferation curves of reseeded PSCs. n = 4 biologically independent experiments; Data presented as means ± SD.; Statistical significance was calculated via two-way ANOVA for main effect of substrate. Western blot analysis of E-cad (**c**), pYAP (at S127) and tYAP levels (**d**) in day-3 EBs formed by reseeding PSCs pre-conditioned on Plain (Plain-Re) and BMS (BMS-Re) substrates. GAPDH was used as a loading control. **e**. Cell cycle progression patterns of day-3 EBs (n = 3 biologically independent experiments; Data presented as means ± SD.; Statistical significance was calculated via two-way ANOVA with Bonferroni’s multiple comparisons test). Source data

To determine how long the mechanical memory and naivety can persist after the removal of BMS, nPSCs were harvested from BMS and reseeded or injected to demonstrate the sustainable effect of BSCR regulation (Fig. 6a). The upregulated E-cad expression and YAP activity of BMS–PSCs remained for at least 15 days after reseeding (Fig. 6b–d). The enhancement in NANOG by BSCR was preserved for at least 10 days (Fig. 6e). The newly formed colonies from BMS–PSCs were more compact, with a higher proliferation rate than those from the plain substrate (Extended Data Fig. 10a,b). Larger embryoid bodies (EBs) were generated from cells preconditioned on BMS (Fig. 6f,g), retaining a higher E-cad level and YAP activity, as well as an accelerated cell cycle progression (Fig. 6h and Extended Data Fig. 10c–e). To exclude the potential influence of EB size on YAP^43^, we generated EBs with controlled dimensions, and a higher YAP activity was still retained in EBs derived from BMS–PSCs (Fig. 6i). Teratoma formation was performed to determine the in vivo proliferation and pluripotency of PSCs. Although cells from both groups were able to form teratomas containing three germ layers, BMS–PSCs were able to develop into teratomas with larger volume (Fig. 6j,k), suggesting their higher development potential/capacity acquired from BMS^40,44^.

## Outlook

In nature, although naive PSCs only transiently exist in the blastocyst, the topographical features of a blastocyst (for example, Epi–TE curvature) may still provide unlimited inspiration. Here learning from nature, we explored the functionality of topographical structures derived from blastocysts on the reversion of PSCs back to a naive state. We established a method to define specific surface topographical maps, and analysed in situ the complexed microstructures on PSC reversion by correlating with time-resolved microscopy images. We concluded that the curvature constraint in a single direction enabled the successful cell reversion through the activation of cell–cell cohesion, intracellular signalling and epigenetic modulation, without necessarily recapitulating the relative radial symmetry of the blastocyst.

We demonstrated that the reverted naivety of PSCs was sustained for at least 10 days after the removal of BMS. The BMS–PSCs processed the higher development potential in generating EBs and teratomas, compared with cells on non-structured substrates.

The BSCR reported here is inspired and simplified from nature, but goes beyond nature, which provides the design criteria for preparing a functional substrate with the purpose to achieve high sustainability in stem cell applications. Importantly, our results indicated the possibility of the BSCR for reverting human PSCs to a more naive state. Although the underlying mechanism of human PSCs should be further explored, the results highlighted the importance of substrate design in the reversion of human PSC naivety and profound applications such as organoid generation, drug screening, disease models and personalized medicine.

## Methods

### Ethical compliance

The animal studies were performed by Experimental Pharmacology and Oncology (EPO), Berlin-Buch, under the approval of the Landesamt für Gesundheit und Soziales Berlin, which reviews animal husbandry based on the German Animal Welfare Act (no. H 0023/09) and in compliance with the EU guideline ‘European convention for the protection of vertebrate animals used for experimental and other scientific purposes (ETS 123)’.

### Determination of mouse blastocyst geometry

The confocal cross views of the preimplantation blastocysts (E4.25–E4.50) from the literature^45–56^ were imported to ImageJ software with the Bio-Formats Importer plugin (National Institutes of Health). The width of the nPSC layer was measured by detecting the line segments between the two endpoints of Epi in contact with polar TE (Supplementary Fig. 1). The distance from the midpoint of the width line to the Epi–TE interface was defined as a height of the outer layer of Epi. The radii of the arcs, curvatures, arc lengths and cell densities of the outer layer of Epi were calculated based on the following formulas: arc radius = width^2^/ (8 × height) + height/2; curvature = 1/arc radius; arc length = 2 × radius × arcsin (width/(2 × radius)); cell density = cell number/arc length. The polar TE layer provides a relatively well-defined curvature of 15 to 62 mm^–1^ (31 ± 8 mm^–1^, means ± standard deviation (s.d.)), which we refer to as the BSCR.

### Defining motifs on cell culture substrates

The scaled three-point approach (Supplementary Methods) was used to measure the curvature of a given point on the BMS in 18 directions. The curvature value of a single direction, *κ*, and the mean curvature values of 18 directions, 〈*κ*〉, were used to define the motif regions on the BMS. Here we define a point as BSCR+ if at least one curvature *κ* for that point is within the BSCR (15 < *κ* < 62 mm^–1^). Next, we distinguished regions that have a positive mean curvature 〈*κ*〉 value (concave) and negative 〈*κ*〉 value (convex). Between both concave and convex regions, we defined a flat region, with some tolerance for positive and negative average curvature values. The regions were defined in detail as follows: convex (〈*κ*〉 < −2.5 mm^−1^), flat (–2.5 < 〈*κ*〉 < 2.5 mm^–1^), concave (〈*κ*〉 > 2.5 mm^–1^).

### Cell culture

The non-viral integrating mouse PSC line (PhiC31, BioCat; reprogrammed from C57BL/6 mouse embryonic fibroblasts (MEFs) with a plasmid encoding OCT4, SOX2, KLF4 and c-Myc) was used for assessing the reversion to naivety. The mouse cell line with NANOG reporter–GFP (iPS-MEF-Ng-492B-4 cells, CiRA, Kyoto University) was used for live-cell tracking. The initial culture of both mouse PSC lines was performed using mitomycin-C-treated feeder MEFs (BioCat) and 0.2% gelatin-coated culture plate, in feeder-dependent PSC culture medium (KnockOut DMEM basal medium containing 15.0% KnockOut Serum Replacement, 1.0% MEM Non-Essential Amino Acids, 1.0% GlutaMAX-I, 0.1% 2-mercaptoethanol and 10 ng ml^–1^ mouse LIF; Life Technologies). Undifferentiated PSCs were isolated using magnetically activated cell sorting technology with Feeder Removal MicroBeads and Pluripotent Stem Cell Isolation Kit (Miltenyi Biotec); PSCs were then maintained in ESGRO-2i/L medium (containing GSK3β and MEK 1/2 inhibitors and 0.5 × 10^6^ U ml^–1^ mouse LIF, Merck Chemicals), and cultured on 9 µg ml^–1^ Cultrex Mouse Laminin (Bio-Techne)-coated six-well tissue culture plates. The PSCs were chemically adapted for at least three passages to the primed-like state by using 1i medium containing GSK3β inhibitor (3 µM CHIR99021, Stemgent) and AXIN stabilizer (2 µM XAV-939, Stemgent)^3^. The 2i/L-PSCs and 1i-PSCs maintained in the tissue culture plates were used as positive and negative controls for naive cells, respectively (Supplementary Fig. 4a). The culture media were changed daily and all the PSCs under the aforementioned culture conditions were used for experiments before reaching passage 50. All the PSCs applied for the experiments detected negative for mycoplasma using MycoFluor Mycoplasma Detection Kit (Thermo Fisher Scientific). The morphology of the PSCs under different culture conditions was monitored; compared with the 2i/L-PSCs, the 1i-PSCs presented primed-like characteristics, including the elongated cell shape and flattened colonies (Supplementary Fig. 4b), as well as decreased levels of NANOG reporter–GFP (the MFI of GFP on plain substrates was set as the threshold) and NANOG proteins (Supplementary Fig. 4c,d).

Human PSC line (BIHi001-A) was a gift from the Stem Cell Core Facility, Berlin Institute of Health. This exogene-integration-free cell line was generated using Sendai virus vectors. Detailed information is available in the hPSCreg database (https://hpscreg.eu/cell-line/BIHi001-A). Human PSCs were maintained on Geltrex (Thermo Fisher Scientific)-coated TCP under feeder-free conditions using Essential 8 medium (Thermo Fisher Scientific). The human PSCs were adapted for ten passages to a naive-like stage using RseT medium (STEMCELL Technologies) in the presence of irradiated CF1 MEFs (Thermo Fisher Scientific) under hypoxic condition (5% O_2_). The RseT–human PSCs were set as a positive control for human PSC analysis.

### Validation of motif function on PSC naivety reversion

The bright-field microscopy images of the BMS were first recognized to be precisely overlaid onto the micro-CT scanning images. The defined BSCR+ area and the concave, flat and convex motifs based on the micro-CT scanning and curvature calculation were then mapped and overlaid with the microscopy images to evaluate the effect of geometrical motifs on cells. In brief, the maximum-intensity-projection confocal laser scanning microscopy (CLSM) images of cells and the motif maps in the same region were imported into Photoshop software (CS6, Adobe Systems), and the motif maps were used as a mask to study the cellular behaviour and parameters on defined areas, including cell size, density, distribution, migration and naive marker level.

### Live-cell tracking

NANOG reporter–GFP integrated in iPS-MEF-Ng-492B-4 cells (CiRA, Kyoto University) was detected by an argon channel (488 nm excitation) to track the living-mouse PSCs^21^. To monitor naivety reversion and cell migration, the real-time fluorescence images of PSCs and bright-field images of the BMS surface in the *Z* stack were acquired using CLSM (Carl Zeiss). PSC expansion on BMS in a single colony were recorded at 20 min intervals using a time-lapse fluorescent microscope IX81 with xCellence/cell^R software (Olympus).

For image analysis, the maximum intensity projection was first performed for all the *Z*-stack CLSM images. The GFP intensity of cells was analysed using ImageJ software (National Institutes of Health). The maximum GFP intensity of the cells on plain substrates was set as a threshold. On BMS, the cells with higher intensity than the threshold were defined as GFP^hi^ naive PSCs. MountainsMap (Digital Surf, Besancon) and Spyder (Python 3.8, Anaconda) were then used to analyse the effect of motifs on GFP^hi^ cell occupancy and naivety level in a time-resolved manner.

### E-cad, RAC1, FAK and YAP activity and inhibition

To examine the distribution and expression of E-cad and F-actin with respect to motifs, cells were fixed, permeabilized and blocked using Image-iT Fixation/Permeabilization Kit (Life Technologies). The anti-E-cad-PE antibody (rabbit monoclonal; 1:10; New England Biolabs) was used for labelling E-cad. F-actin was detected using ActinRed 555 ReadyProbes Reagent (Life Technologies). The CLSM images of the samples were then overlaid with the motif maps following the method shown in Supplementary Fig. 4.

To quantify the E-cad level, 1 × 10^6^ cells were freshly harvested from the substrates at the indicated time points and then immediately processed for anti-E-cad-PE antibody (rabbit monoclonal; 1:50; New England Biolabs) staining in the dark at room temperature for 30 min. The labelled cells were measured by flow cytometry (MACSQuant, Miltenyi Biotec) and analysed using FlowJo software (version 10.4, Tree Star Inc.).

The concentration of phosphorylated FAK and total FAK in the cell extract were measured using the pFAK (pY397)/tFAK enzyme-linked immunosorbent assay (ELISA) kits (Thermo Fisher Scientific). The same amount of total protein determined using a BCA protein assay kit (Thermo Fisher Scientific) was applied for ELISA.

For the quantification of YAP and AMOTL1 phosphorylation levels in PSCs, day-3 cells growing on different substrates were harvested, fixed with 4% paraformaldehyde and permeabilized with prechilled 90% methanol for 30 min on ice, then stained with anti-YAP-Alexa Fluor 647 and anti-phospho-YAP (S127) (rabbit monoclonal; 1:50; New England Biolabs) or anti-phospho-AMOTL1 (S262) antibodies (rabbit polyclonal; 1:100; Covalab Biotechnology) for 45 min. The cells were then stained with secondary anti-rabbit IgG (H + L)-Alexa Fluor 647 and anti-rabbit IgG (H + L) Alexa Fluor 488 antibodies (goat polyclonal; 1:500; New England Biolabs) for 30 min, followed by flow cytometry analysis.

Inhibition experiments were performed by treating the cells using the RAC1 activity inhibitor NSC 23766 (50 µM; Bio-Techne), the selective FAK inhibitor PF-573228 (10 µM; Sigma-Aldrich) and the YAP activation inhibitor Verteporfin (20 nM; Sigma-Aldrich). For E-cad neutralization, cells were treated with E-cad antibody (25 µg ml^–1^; Merck) for 24 h before harvesting.

### ChIP PCR

ChIP was performed using the SimpleChIP Plus Enzymatic Chromatin IP Kit (New England Biolabs) following the given instruction. ChIP employed anti-H3, anti-H3K27me3 and anti-H3K4me3 antibodies (rabbit monoclonal; 1:50; New England Biolabs) with magnetic beads. After washes, the samples were eluted and treated with RNase and proteinase K overnight. The released DNA was used for real-time PCR quantification. Enrichment was calculated relative to the input DNA data and expressed as per cent: input = 10% × 2^(CT 10% input sample – CT ChIP sample)^ and then normalized to the total H3 enrichment level. Normal rabbit IgG (1:50; New England Biolabs) was used as an isotype control. The oligonucleotide sequences were *Nanog* promoter forward primer 5′-GGAGAATAGGGGGTGGGTAG and reverse 5′-CAGCCTTCCCACAGAAAGAG. *Zic2* promoter forward primer 5′- TGGACTCTTCTCCTCCTCCA and reverse 5′-GGTGGAAATACTGGCGACTG. *Pou5f1* distal enhancer forward primer 5′- GGCTGCAGGCATACTTGAAC and reverse 5′-AGGGCAGAGCTATCATGCAC.

### Stability of naivety of BMS–PSCs

PSCs were cultured on plain substrate and BMS for 5 days and then harvested and reseeded to evaluate the stability of naivety. On laminin-coated TCP, the cells were seeded at a density of 2 × 10^4^ cm^–2^ and cultured in a monolayer for four passages, followed by anti-E-cad-PE (rabbit monoclonal; 1:10; New England Biolabs), anti-YAP-Alexa Fluor 488 (rabbit monoclonal; D8H1X; 1:100; New England Biolabs) and anti-NANOG-APC (recombinant human monoclonal; 1:11; Miltenyi Biotec) staining. The samples were then examined with a CLSM (LSM780, Carl Zeiss) with Zen 2012 software (Carl Zeiss) and flow cytometry.

EBs were obtained by reseeding the cells (2 × 10^4^ cm^–2^) on uncoated TCP, and cultured in complete KnockOut Serum Replacement EB medium (Thermo Fisher Scientific). To form EBs with uniform dimensions, a 96-well V-bottom plate (Sigma-Aldrich) was used. The generated EBs were fixed, permeabilized and incubated with anti-YAP (rabbit monoclonal; D8H1X; 1:100; New England Biolabs) overnight at 4 °C. The secondary anti-rabbit IgG (H + L)-Alexa Fluor 647 or anti-rabbit IgG (H + L)-Alexa Fluor 488 antibodies (goat polyclonal; 1:500; New England Biolabs) were used for labelling. After 1 h of culture at room temperature, the fluorescence was detected and imaged by LSM780 (Carl Zeiss) with Zen 2012 and Zen 2.3 software (Carl Zeiss). The YAP phosphorylation level was examined using western blot (Supplementary Methods).

The teratoma formation experiment was performed by EPO and carried out in accordance with the German Animal Protection Law and approved by the local responsible authorities. EPO complies with the EU guideline ‘European convention for the protection of vertebrate animals used for experimental and other scientific purposes (ETS 123)’. Animals were handled according to the ‘Regulation on the protection of experimental scientific purposes or other purposes used animals’. Compliance with the above rules and regulations is monitored by the Landesamt fuer Gesundheit und Soziales, which is the responsible regulatory authority monitoring the animal husbandry based on the German Animal Welfare Act. Approval (code H 0023/09) was given after careful inspection of the site including bedding, feeding and water, ventilation, temperature and humidity, and cleaning and hygiene concepts. The day-5 PSCs derived from plain substrates and BMSs were subcutaneously injected into eight-week-old male NSG immunodeficient mice (1 × 10^6^ PSCs for each mouse). The teratoma diameter measurements were performed twice weekly until the end day of the study when teratoma reached 1.5 cm^3^. Teratoma volume was calculated using the following formula: volume = width^2^ × length/2. The dissected teratomas were cryoconserved or formalin fixed for haematoxylin and eosin staining.

### Statistical analysis

Statistical analysis for biological evaluation was performed using Prism 8.0 (GraphPad) software. Comparisons between two experimental groups or the same group with and without inhibitors were analysed using two-tailed unpaired sample Student’s *t*-tests. Before performing the Student’s *t*-test, the homogeneity of variance for the two groups was verified by an F test. The differences among three or more independent groups were analysed using one- or two-way analysis of variance (ANOVA) followed by Bonferroni’s multiple comparisons test. In particular, the two-way ANOVA was applied to analyse the individual effects of motifs and BSCR counts on regulating cell distribution, proliferation and naive markers, as well as their overall effects over time. The sample sizes for each experimental group and the statistical test used to determine significances among groups are reported in the figure legends. Briefly, for the following in vitro and in vivo studies, statistical analyses were applied to biologically independent samples (separate batches/plates of cells, cell lysates and immunodeficient mice) across plain substrate and BMS: quantification of naive-pluripotency-associated markers, E-cad, FAK, YAP, histone H3 modification at naive gene loci, cell death, migration, cell cycle sub-phases, cell expansion and teratoma formation. For image-based analysis with ImageJ software (version 2.0.0-rc-65/1.52b, National Institutes of Health), the numbers of images, cell nuclei and colonies were indicated in the corresponding figure legends. In all the cases, *P* values of less than 0.05 were considered statistically significant and the exact *P* values are indicated in the figures. Unless indicated otherwise, the quantification data are presented as means ± s.d.

### Reporting summary

Further information on research design is available in the Nature Portfolio Reporting Summary linked to this article.

## Online content

Any methods, additional references, Nature Portfolio reporting summaries, source data, extended data, supplementary information, acknowledgements, peer review information; details of author contributions and competing interests; and statements of data and code availability are available at 10.1038/s41563-024-01971-4.

## Supplementary information

Supplementary Information Supplementary Figs. 1–8, Tables 1–3, captions for Videos 1 and 2, Methods and references.

Reporting Summary

Supplementary Video 1 Migration of whole population of PSCs on BMS from day 0 (12 h) to day 1 (25 h). The iPS-MEF-Ng-492B-4 mouse PSC line with NANOG reporter–GFP was used for live-cell tracking. Images were recorded within 60 min intervals. The outlines of BSCR+ areas are illustrated with dashed yellow lines. The video was exported and played at 5 fps. Scale bar, 100 μm.

Supplementary Video 2 Migration of NANOG-GFP^hi^ naive PSCs on BMS from day 1 (36.0 h) to day 3 (73.5 h). The iPS-MEF-Ng-492B-4 mouse PSC line with NANOG reporter–GFP was used for live naive PSCs tracking. GFP^hi^ naive PSCs were discriminated using a fluorescence threshold based on the plain control. Images were recorded within 90 min intervals. The outlines of BSCR+ areas are illustrated with dashed yellow lines. The video was exported and played at 5 fps. Scale bar, 100 μm.

## Source data

Source Data Fig. 1 Statistical source data.

Source Data Fig. 2 Statistical source data.

Source Data Fig. 3 Statistical source data.

Source Data Fig. 4 Statistical source data.

Source Data Fig. 5 Statistical source data.

Source Data Fig. 6 Statistical source data.

Source Data Extended Data Fig. 1 Statistical source data.

Source Data Extended Data Fig. 2 Statistical source data.

Source Data Extended Data Fig. 4 Statistical source data.

Source Data Extended Data Fig. 6 Statistical source data.

Source Data Extended Data Fig. 7 Statistical source data.

Source Data Extended Data Fig. 8 Statistical source data.

Source Data Extended Data Fig. 9 Statistical source data.

Source Data Extended Data Fig. 10 Statistical source data.

Uncropped Blots for Extended Data Fig. 10 Unprocessed western blots.

## Data Availability

All data supporting the findings of this study are available within the Article and its Supplementary Information. Source data are available via Figshare at 10.6084/m9.figshare.25827706 as well as are provided with this Article.

## Code availability

The custom code written for this paper uses Python 3.8 and the accompanying packages numpy, numpy-stl, matplotlib, scipy and pandas were used to analyse the different substrates mentioned in the paper. The code scripts are available via Zenodo at 10.5281/zenodo.10732261 (ref. ^57^).
