# Supplementary material for: Substrates mimicking the blastocyst geometry revert pluripotent stem cell to naivety
Source: Nat Mater. 2024 Aug 12;23(12):1748–58. doi: 10.1038/s41563-024-01971-4 (PMC11599042; doi:10.1038/s41563-024-01971-4)
Supplement: Supplementary file 1 — Supplementary Figs. 1–8, Tables 1–3, captions for Videos 1 and 2, Methods and references. [file 41563_2024_1971_MOESM1_ESM.pdf]

nature materials

Supplementary information

https://doi.org/10.1038/s41563-024-01971-4

Substrates mimicking the blastocyst geometry revert pluripotent stem cell to naivety

In the format provided by the authors and unedited

Table of Contents

Supplementary Videos Legends

- **Supplementary Videos 1.** Migration of whole population of PSCs on BMS from day 0 (12 h) to day 1 (25 h).
- **Supplementary Videos 2.** Migration of NANOG-GFP^{hi} naïve PSCs on BMS from day 1 (36 h) to day 3 (73.5 h).

Supplementary Methods

- Fabrication of BMS
- Fabrication of microbowl and microgroove substrates
- Micro computed tomography (micro-CT) analysis of BMS
- *Scaled three-point* approach for curvature measurement
- Laminin (LMN) coating and characterization
- Confocal laser scanning microscopy (CLSM)
- Flow cytometry
- Real-time PCR Array
- Western blot
- Alkaline phosphatase (AP) staining
- Cell proliferation assay
- RAC1 activation G-LISA
- pMLC2 imaging and quantification
- Collagen matrix-based contractility assay

Supplementary Figures

- **Supplementary Fig. 1.** Characterization of pre-implantation blastocysts
- **Supplementary Fig. 2.** Motif and curvature distribution of BMS surfaces
- **Supplementary Fig. 3.** Scaled three-point approach for curvature measurement
- **Supplementary Fig. 4.** Correlative analysis integrating geometrical and biological information.
- **Supplementary Fig. 5.** Characterization of PSCs with naïve and primed-like states
- **Supplementary Fig. 6.** Characterization of laminin (LMN) coated Plain and BMS substrates.
- **Supplementary Fig. 7.** Threshold determination for the confocal images of BMS-PSCs
- **Supplementary Fig. 8.** Image processing and analysis for naïvety reversion and cell proliferation.

Supplementary Tables

- **Supplementary Table 1.** Comparism of surface parameters between metal molds and BMS according to DIN EN ISO 25178
- **Supplementary Table 2.** Parameters of microbowls
- **Supplementary Table 3.** Parameters of microgrooves

Supplementary References

Supplementary Videos Legends

Supplementary Video 1. Migration of whole population of PSCs on BMS from day 0 (12 h) to day 1 (25 h). The iPS-MEF-Ng-492B-4 mouse PSC cell line with NANOG-reporter GFP was used for live cell tracking. Images were recorded within 60 min intervals. The outlines of BSCR+ areas were illustrated with yellow dash lines. The movie was exported and played at 5 fps. Scale bar, 100 µm.

Supplementary Video 2. Migration of NANOG-GFP^{hi} naïve PSCs on BMS from day 1 (36 h) to day 3 (73.5 h). The iPS-MEF-Ng-492B-4 mouse PSC cell line with NANOG-reporter GFP was used for live naïve PSCs tracking. GFP^{hi} naïve PSCs were discriminated using a fluorescence threshold based on the Plain control. Images were recorded within 90 min intervals. The outlines of BSCR+ areas were illustrated with yellow dash lines. The movie was exported and played at 5 fps. Scale bar, 100 µm.

Supplementary Methods

Fabrication of BMS

Four metal cylinders (steel type 1.2210, 60 HRC) were prepared using the electrical discharge machining (EDM). For the spark erosion process an EDM machine of model Gantry Eagle 500 (OPS-INGERSOLL Funkenerosion GmbH, Burbach, Germany) was applied utilizing an electrode type graphite EDM 200 (Poco Graphite SARL, Entegris, Inc., Limonest, France) and IonoPlus IME MH (Oelheld GmbH innovative fluid technology, Stuttgart, Germany) as dielectric medium. The machine has an adaptive current pulse generator Eagle Powertec Fine Finishing generator PT60 with maximum machining power 60 A. The estimated average energy per spark is 5.4 - 47.7 µJ. Machine parameters were used as following: work piece material: Steel; electrode material: Grs Graphit standard; electrode geometry: Standard (free form); engagement area: 78.0 mm^2; cross section area: 78.0 mm^2; erosion depth: 25 mm; and priority: low-wear. Each metal cylinder was machined for 30 minutes to achieve a surface finish grade M45 according to the guideline VDI 3400 surface finish grade definition [1]. This corresponds to a surface roughness of Ra ~ 17.8 µm and a maximum distance between the highest peak and lowest valley in the whole sampling of Rt ~ 110.0 µm. Electrode-Typ 1 was used with an undersize distance of 0.3 mm. With this roughness, blastocyst relevant dimensions were reached. Finally, the eroded metal cylinders were intensively purified utilizing PowerCleaner 200 (Bio-Circle Surface Technology GmbH, Gütersloh, Germany).

Polystyrene (PS, type 158K, BASF, Germany) with a number average molecular weight of M_n = 109 000 g mol^{-1} was used without any further purification. Processing of the BMS polymer inserts fitting into standard 24-well tissue culture plates was performed by injection molding using an injection molding automat (Alrounder 270U, Arburg Corp., Münsingen, Switzerland) equipped with a custom made mold (Dreuco Formenbau GmbH, Berlin, Germany). In a two-stage process a volume 6.5 cm^3 PS was injected with injection rates of 14 and 20 cm^3·s^{-1} and pressures of 1200 and 1800 bar. The applied temperatures were 35 °C (heating zone I), 210 °C (heating zone II) and 230 °C (heating zone III and IV), while the mold was kept at 30 °C. The molds consisted of the above mentioned four individually surface-finished metal cylinders, allowing the parallel fabrication of four inserts (BMS1-

BMS4) with an upper inner diameter of 12.4 mm, a lower inner diameter of 10.5 mm, a height of 16.8 mm, and a wall thickness of 1 mm.

Prior to use, all prepared PS inserts were sterilized by ethylene oxide gas sterilization (gas phase: 10% ethylene oxide, 54 °C, 65% relative humidity, 1.7 bar, 3 hours of gas exposure time and 21 hours of aeration phase).

Fabrication of microbowl and microgroove substrates

PS based microbowl (μB) and microgroove (μG) substrates were prepared to study the effect of regular micro-structure on PSCs.

Acid washed glass beads (Sigma-Aldrich Chemie GmbH, Taufkirchen, Germany) with diameters of ~75, 150~210, and ~250 μm were used to prepare the μB-substrate. These glass beads were first functionalized with silanization solution (1% dichlorodimethylsilane in ethanol, Sigma) and then rinsed with ethanol before air-dried in the hood. Polyvinyl alcohol (PVA) was dissolved as 5% (w/v) solution in de-ionized water, and 5 mL of the solution was placed into a 35 mm petri dish. Silanized glass beads were then gradually dropped onto the meniscus, which floated on the liquid-air interface due to hydrophobicity, and spontaneously assembled into a single, closely packed layer under ultrasound for 30 minutes.

The μG-substrates were produced by photolithography methods. 2-inch silicon wafers were spin-coated with 2 mL of ma-P 1275G positive photoresist (Micro resist technology GmbH, Berlin, Germany) at 500 rpm for 60 s, followed by 1 hour evaporation and a three-stage baking step at 70 °C, 90 °C, 115 °C for 5 minutes each. Photomasks designed by Autodesk software were printed at 128k dpi (JD Photo Data company, Hitchin, UK), placed on the coated wafer, and exposed for 80 s to UV-light irradiation (mercury arc lamp 365 nm, intensity 114 mW/cm^2; Solar simulator system, Abet technology) using a UV filter (cut-on 365 ± 7 nm, Laser Components GmbH, Olching, Germany). Finally, the exposed wafers were post-baked at 100 °C for 30s and developed in mr-D 526/S developer (Micro resist technology GmbH, Berlin, Germany) with gentle shaking. Finally, the developed silicon wafer was heated at 105 °C for 5 minutes to allow the reflow process, which converts the rectangular cross-sectional shape of photoresist to a curved shape.

The poly(dimethylsiloxane) (PDMS) soft mold replicate of the assembled glass beads or structured silicon wafer was synthesized from a precursor mixture of 90 wt% prepolymer Sylgard 184 and 10 wt% curing agent (Dow Corning Corp., Midland, MI) by curing at 80 °C for 24 h. The achieved PDMS mold was applied as negative template for producing a second PDMS soft mold comprising microprotrusions following the aforementioned procedure. For preparing smooth surface, the PDMS soft mold was replicated from smooth silicon wafer.

Finally, the PS substrates with μB and μG microstructures were obtained by soft-lithography according to the reference[2], using the prepared PDMS molds. Prior to use, the microstructured PS substrates were sterilized by ethylene oxide gas sterilization (gas phase: 10% ethylene oxide, 54 °C, 65% relative humidity, 1.7 bar, 3 hours of gas exposure time and 21 hours of aeration phase).

Micro computed tomography (micro-CT) analysis of BMS

An X-ray micro-CT of ProconXray GmbH (Germany) was used for characterization of surface texture of BMS inserts. Recording of grey shadow images was realized with an X-ray output of 40 kV and 0.2 mA at small spot. The distance between X-ray source and sample resulted in a voxel dimension of 9.1 μm edge length. The integration time was 750 ms at averaging of 6 images. Overall 960 images, recorded with an angle distance of 0.375 degrees, were used for reconstruction of 3D images. The BMS inserts were oriented perpendicular to the measurement beam. The post treatment of 3D-images was carried out with MAVI software (Fraunhofer Society, Germany). The reconstructed 3D-images (example showed in

Supplementary Fig. 2a) were filtered with a binomial filter stage 19 and binarized by the "Otsu" algorithm.

For 3D surface topography analysis of the PS inserts, the reconstructed surfaces from micro-CT (stored as .stl-files) were analyzed using MountainsMap® software (Digital Surf, Besancon, France) corresponding to EN ISO 25178. Initially a circular shape was cut out from the bottom of the insert, with the side walls removed, and thereafter an operator for leveling by a least square method was applied. The height-maps of the four BMSs with a diameter of 10.8 mm are shown in **Supplementary Fig. 2b**. The images show round-shaped elevations and hollows, confirming the absence of sharp edges and vertical or horizontal planar areas. The x,y,z-data on a spacing grid of 15 μm in x- and y-direction were used for further curvature evaluation using Origin software (Originlab, USA), Matlab (Mathworks, USA), Spyder (Python 3.6, Anaconda Inc. USA).

***Scaled three-point* approach for curvature measurement**

The method we have used in this paper we label scaled three-point curvature and is based on Heron's formula. On a surface S for any given point p, curvature is analyzed around the point itself 360 degrees (**Fig. 1b**). This is done by giving a direction in the form of a vector, v, then two other points are selected by going in the positive direction of the vector and the negative direction of the vector. Once the three points are selected, a system of equations can be written to solve for a circle that touches all three points. The inverse of the radius is the curvature for that point p in the direction v. This is then repeated for n directions for each point. We chose to compute curvature, κ, at each 10 degree increment, yielding 36 curvatures. However, due to symmetry only 18 are unique. The magnitude of vector, v, is the scale, with which the curvature was analyzed. The magnitude for v was set to 30 μm, as this reflected half the width of a Epi which is ~60 μm (**Supplementary Fig. 3**).

Laminin (LMN) coating and characterization

To facilitate mouse PSC attachment, Plain and BMS substrates were pretreated with 200 μl of 9 μg/ml Cultrex Mouse Laminin (Bio-Techne GmbH). Anti-laminin antibody (rabbit monoclonal; 1:100; Abcam) was used for LMN immunostaining. The distribution of LMN on different substrates (20 images) and in different motif areas (10 images) was quantified via the confocal image-based analysis of LMN intensity using imageJ software (National Institutes of Health). The LMN formed a homogeneous layer without strong difference in coating density on BMS and Plain control, as well as between different motifs. The amounts of LMN in the coating solution at day 1 and released in PBS from day 2-5 on different substrates were quantified using LMN ELISA Kit (abcam). Three independent experiments were included. The LMN solution was collected daily and the LMN adsorption amount was calculated by subtracting the soluble LMN from total LMN amount at the previous day. The amount of LMN remained to be constant for 5 days, indicating the stability of the LMN layer. Further, the advancing and receding contact angles of LMN coated and uncoated substrates were measured with drop shape analyzer (DSA 100, Krüss GmbH) using the captive bubble method. All samples were pre-conditioned for 24 hour in deionized water at ambient temperature for equilibration. 10 measurements for advancing and receding angles on five different locations were performed for each sample. LMN coating increased the wettability of the substrates.

Confocal laser scanning microscopy (CLSM)

To measure the cell diameters, the cytosol of live PSCs was stained with 1 μM CellTrace™ Far Red (Thermo Fisher Scientific Inc.) for 15 min at 37 °C. The dead or dying cells on BMS were detected with 1 μg/ml propidium iodide solution (Miltenyi Biotec.).

To investigate the effect of BSCR on PSC proliferation, iPSCs (iPS-MEF-Ng-492B-4 cells with integrated NANOG reporter-GFP, CiRA, Kyoto University, Japan) with different initial state (Primed, naïve, and mixed population (naïve:primed = 1:4 in number)) were seeded on BMS substrate as well as Plain surface at a seeding density of 2×10^4 cells/cm^2. Subsequently, primed and mixed PSCs were cultured in 1i medium, while naïve PSCs were cultured in 2i/L medium. Ki67 and DAPI staining were carried out at specified time points for comprehensive analysis.

For intracellular and intranuclear imaging, cells were fixed, permeabilized and blocked using Image-iT Fixation/Permeabilization Kit (Life Technologies). Click-iT™ Plus TUNEL Assay Kit (Thermo Fisher Scientific Inc.) was used for *in situ* apoptosis detection with Alexa fluor 647 dyes. Cells were stained with primary antibodies overnight at 4 °C and were then incubated with corresponding Alexa fluor 488 conjugated anti-mouse or rabbit IgG (H+L) secondary antibodies (1:800; Thermo Fisher Scientific Inc.), anti-mouse IgG (H+L)-Alexa fluor 647 secondary antibody (1:500; Thermo Fisher Scientific Inc.) and anti-rabbit IgG (H+L)-Alexa fluor 633 secondary antibody (1:800; Thermo Fisher Scientific Inc.) for 1h at room temperature in the dark. The following primary antibodies were used: anti-NANOG (rabbit polyclonal; 1:10; Thermo Fisher Scientific Inc.), anti-STELLA (mouse monoclonal; 1:50; Merck KGaA) and anti-TBX3 (rabbit polyclonal; 1:200; Thermo Fisher Scientific Inc.), anti-Ki67 antibody (rabbit monoclonal 1:400; New England Biolabs GmbH) and rabbit IgG isotype control antibody (Thermo Fisher Scientific Inc.). The viable and fixed cell nuclei were stained with Hoechst 33342-based NucBlue Live ReadyProbes (Life Technologies) and DAPI (Thermo Fisher Scientific Inc.), respectively. CLSM imaging was carried out using LSM 780 (Carl Zeiss). The stitched tile scanning (7 mm x 7 mm) and the local scanning (1.4 mm x 1.4 mm and 1.4 mm x 0.7 mm) of z-stack images of NANOG, STELLA and the bright field view of PSCs on BMS at indicated time points were recorded. The cross views were reconstructed using Zen 10 (Carl Zeiss Microscopy GmbH) or "dynamic resliced" function of ImageJ (National Institutes of Health). Maximum intensity projection was performed for all the z-stack image series of cells on Plain and BMS. The maximum fluorescence intensity of NANOG and STELLA in cells grown on Plain substrates were set as thresholds. The CLSM images of $NANOG^{hi}$ and $STELLA^{hi}$ cells used for the following motif and BSCR analysis were obtained by applying the thresholds, respectively (**Supplementary Fig. 7**). To study the cell proliferation activity of PSCs, a set of fluorescence images, including GFP, Ki67, nuclei, and bright-field images in Z-stack, was acquired. A maximum intensity projection was subsequently carried out for analysis. The threshold for the GFP filter was set using the maximum GFP intensity of 1i-PSCs on the Plain substrate, discriminating between GFP^{hi} and GFP^{low} cells. Ki67+ cells were defined using the maximum intensity of the Ki67-isotype control. The BSCR mask, derived from micro-CT analysis, was employed to differentiate between BSCR+ and BSCR- areas. The GFP^{hi}-cell covered area within the BSCR+ region served to create the mask for GFP^{hi}/BSCR+. For precise cell identification, Otsu's Local Thresholding and Watershed Separation were applied through image segmentation. ImageJ Macro programming (National Institutes of Health) was utilized for the automation of image analysis, as illustrated in **Supplementary Fig. 8**.

Flow cytometry

To analyze the mouse PSC naïvety, $1\text{x}10^6$ cells were freshly harvested at indicated time points, then immediately processed for flow cytometry analysis of surface markers. The fixed and permeabilized cells were used for analyzing of intracellular proteins. Cells were incubated directly with anti-stage-specific embryonic antigen (SSEA) 1-PE (mouse monoclonal; 1:50; Thermo Fisher Scientific Inc.) and anti-SSEA-4-Alexa fluor 647 (mouse monoclonal; 1:10; BD Biosciences), anti-NANOG-APC (REAfinity recombinant human IgG;

1:11; Miltenyi Biotec.), anti-OCT4-PE (REAfinity recombinant human IgG; 1:11 Miltenyi Biotec.), anti-STELLA (mouse monoclonal; 1:50; Merck KGaA) and anti-ZIC2 (rabbit monoclonal; 1:600; abcam) antibodies in dark at room temperature for 30 minutes. The primary antibody solution of anti-STELLA and anti-ZIC2 was removed. Cells were then incubated with anti-mouse and anti-rabbit IgG (H+L)-Alexa fluor 647 antibodies (goat polyclonal; 1:500; New England Biolabs GmbH) for another 30 minutes, respectively. Labeled cells were measured by flow cytometry (MACSQuant, Miltenyi Biotec.) and analyzed using "Flowjo" software (Tree Star Inc.). For human PSC measurement, the following antibodies were used: anti-CD7-APC (REAfinity recombinant human IgG; 1:50; Miltenyi Biotec.), anti-CD24-APC (REAfinity recombinant human IgG; 1:50; Miltenyi Biotec.), anti-CD57-FITC (mouse IgM monoclonal; 1:50; Miltenyi Biotec.), anti-CD90-FITC (REAfinity recombinant human IgG; 1:50; Miltenyi Biotec.), anti-E-Cadherin-PE (rabbit monoclonal; 1:50; New England Biolabs GmbH) for living cells, and anti-NANOG-APC (REAfinity recombinant human IgG; 1:11; Miltenyi Biotec.) for fixed/permeabilized cells.

To analyze the alterations in cell cycle kinetics, the PSCs were seeded with the density of 1.5×10^4 cells/cm^2. Day 3 PSCs were harvested using accutase (Merck KGaA) and fixed overnight with cold 70% ethanol at 4 °C and subsequently stained with FxCycle PI/RNase Staining Solution (Life Technologies) at room temperature for 30 minutes. DNA content was estimated by flow cytometry, and the fractions of cells in different phases were analyzed using "ModFit LT" software (Verity Software House). The same procedure was applied for EBs derived from PSCs pre-conditioned on different substrates. Accumax (Merck KGaA), Embryoid Body Dissociation Kit and gentleMACS Dissociator (Miltenyi Biotec.) were used for EB detachment and dissociation. Single cell samples were applied for all the flow cytometric measurements (MACSQuant, Miltenyi Biotec.).

Real-time PCR Array

RNA was extracted and purified from the day 3 PSCs using the RNeasy Mini Kit (QIAGEN GmbH), and was subsequently transcribed into cDNA with the RT2 First Strand Kit according to the manufacturer's protocol (QIAGEN GmbH). Real-time PCR array analysis was performed using mouse embryonic stem cell and mouse Hippo signaling pathway RT2 Profiler PCR Array microplates (QIAGEN GmbH) on a StepOne Plus Real-time PCR System (Applied Biosystems). Data were uploaded and analyzed with free online RT2 Profiler PCR Array Data Analysis Web software (QIAGEN GmbH). The cut-off was set as CT (threshold cycle) values higher than 35, which considered as absence of gene expression. The CT value of mouse heat shock protein 90 kDa alpha, class B member 1 (Hsp90ab1) housekeeping gene in each sample was used to normalize the ΔCT values of target genes (ΔCT = CT, target - CT, housekeeping). The gene expression level was expressed as $2^{-\Delta CT}$, and the fold change between two samples (Sample 2/Sample 1) was expressed as $2^{-\Delta\Delta CT}$ (ΔΔCT = ΔCT, target, Sample 2 - ΔCT, target, Sample 1).

Western blot

2×10^4/cm^2 pre-conditioned PSCs from Plain and BMS were reseeded to the 6-well suspension culture plate to form EBs. At day 3, EBs were lysed at room temperature for 10 minutes with M-PER Mammalian Protein Extraction buffer containing a mixture of 1× Halt Protease & Phosphatase Inhibitor Cocktail (Thermo Fisher Scientific Inc.). The protein concentration in the supernatant was determined using a BCA Protein Assay Kit (Thermo Fisher Scientific Inc.). 20 µg of total protein from each sample was loaded to the AnykD precast SDS-PAGE gel (Bio-Rad Laboratories GmbH) and electrophoresis was performed at 150 V. When the protein dye reached the bottom of the gel, the protein was transferred onto the nitrocellulose membrane (Merck KGaA) at a constant current of 200 mA for 1 h. Then,

the membrane was blocked with Odyssey Blocking Buffer (LI-COR Biosciences) and stained with anti-E-cad (mouse monoclonal; 1:500; Thermo Fisher Scientific Inc.), anti-phospho-YAP (S127), anti-YAP and anti-GAPDH antibodies (rabbit monoclonal; 1:1,000; New England Biolabs). Finally, the primary antibodies bound to the membrane were detected with IRDye 800CW secondary antibody and visualized using an Odyssey Infrared Imaging System with Odyssey Infrared Imaging software (ver 3.0.25, LI-COR Biosciences). The experiment was performed using three independent cell preparations and the protein level was quantified by analyzing the density of bands with ImageJ software (National Institutes of Health).

Alkaline phosphatase (AP) staining

Stemgent Alkaline Phosphatase Staining Kit II (Miltenyi Biotec) was applied for AP staining of PSCs. Cells were fixed with Fix Solution at room temperature for 5 minutes. The fixed cells were washed with PBST and incubated with freshly prepared AP substrate solution in the dark at room temperature for 10 minutes. The reaction was stopped by washing the wells twice with PBS. Cells were then covered with PBS or mounting medium to prevent drying and stored at 4°C for microscopy.

Cell proliferation assay

The PSCs were seeded with the density of 1.5×10^4 cells/ cm^2 on Plain and BMS substrates. The relative number of cells at day 1, 3, 5 was determined using the Cell Counting Kit-8 (CCK-8, Dojindo Molecular Technologies). In brief, old medium was replaced with 300 µl of fresh medium, followed by adding 30 µl CCK-8 solution. After 2 h of incubation at 37 °C, 100 µl medium/CCK-8 mixture was transferred into a transparent 96-well plate, and the absorbance was measured at a wavelength of 450 nm (reference: 650 nm) using a microplate reader (Infinite 200 PRO, Tecan Group Ltd.) with Magellan software (Tecan Group Ltd.). The cell number was calculated via a standard curve, which was produced by measuring a series of samples with known cell number.

RAC1 activation G-LISA

Cells were washed and lysed in ice-cold cell lysis buffer provided in G-LISA RAC1 activation assay kit (Biomol GmbH) and immediately clarified by centrifugation at 10,000 × g, 4°C for 1 minutes. The protein concentration was determined using Precision Red Advanced Protein Assay Reagent. The powder immobilized in RAC1-GTP binding well strip was dissolved on ice for 20 minutes and 50 µl of concentration-equalized lysates, RAC1 positive and buffer blank control were then applied and incubated on a cooled orbital microplate shaker at 400 rpm, 4°C for 30 minutes. Wells were washed twice and incubated with 200 µl of antigen presenting buffer for 2 minutes. 50 µl of 1:50 diluted anti-RAC1 antibody was loaded and incubated at 400 rpm for 45 minutes. 50 µl of secondary antibody was added and incubated at 400 rpm for 45 minutes. 50 µl of the freshly prepared HRP detection reagent (reagent A and B in equal volumes) was loaded and incubated for 20 minutes. After 50 µl of HRP stop buffer was applied to each well. The absorbance was measured at 490 nm using the Tecan Infinite 200 PRO microplate reader (Tecan Group Ltd.).

pMLC2 imaging and quantification

Day 3 PSCs were directly fixed on culture substrates or detached and harvested followed by a fixation with 4% paraformaldehyde. 0.5% Triton X-100 was used for permeabilization. Cells were then stained with the primary anti-phospho-myosin light chain 2 (S19) antibody (mouse monoclonal; 1:100; New England Biolabs GmbH) followed by the staining of the anti-mouse IgG (H+L)-Alexa fluor 647 secondary antibody (goat; 1:500; New England

Biolabs GmbH). The fluorescent intensity of pMLC2 in Concave+ and Concave- motifs was measured by LSM780 microscope (Carl Zeiss). The overall level of pMLC2 in cell grown on substrates was detected by MACSQuant flow cytometer (Miltenyi Biotec.). Data analysis was performed using "Flowjo" software (Tree Star Inc.).

Collagen matrix-based contractility assay

The working solution of collagen gel was freshly prepared and kept on ice according to the user manual of Cell Contraction Assay Kit (BioCat GmbH). Single cell suspension (2×10^6 cells/ml) was collected and mixed with collagen gel (1:4 vol/vol). The mixture was then loaded and solidified in the culture plate at 37°C for 1 hour. At day 3, the stressed gel was gently released from the side of culture well with a sterile spatula. 8 hours after releasing, the covered area change of the gel (% of initial covered area) was captured by microscope and measured by ImageJ software (National Institutes of Health).

Supplementary Figures

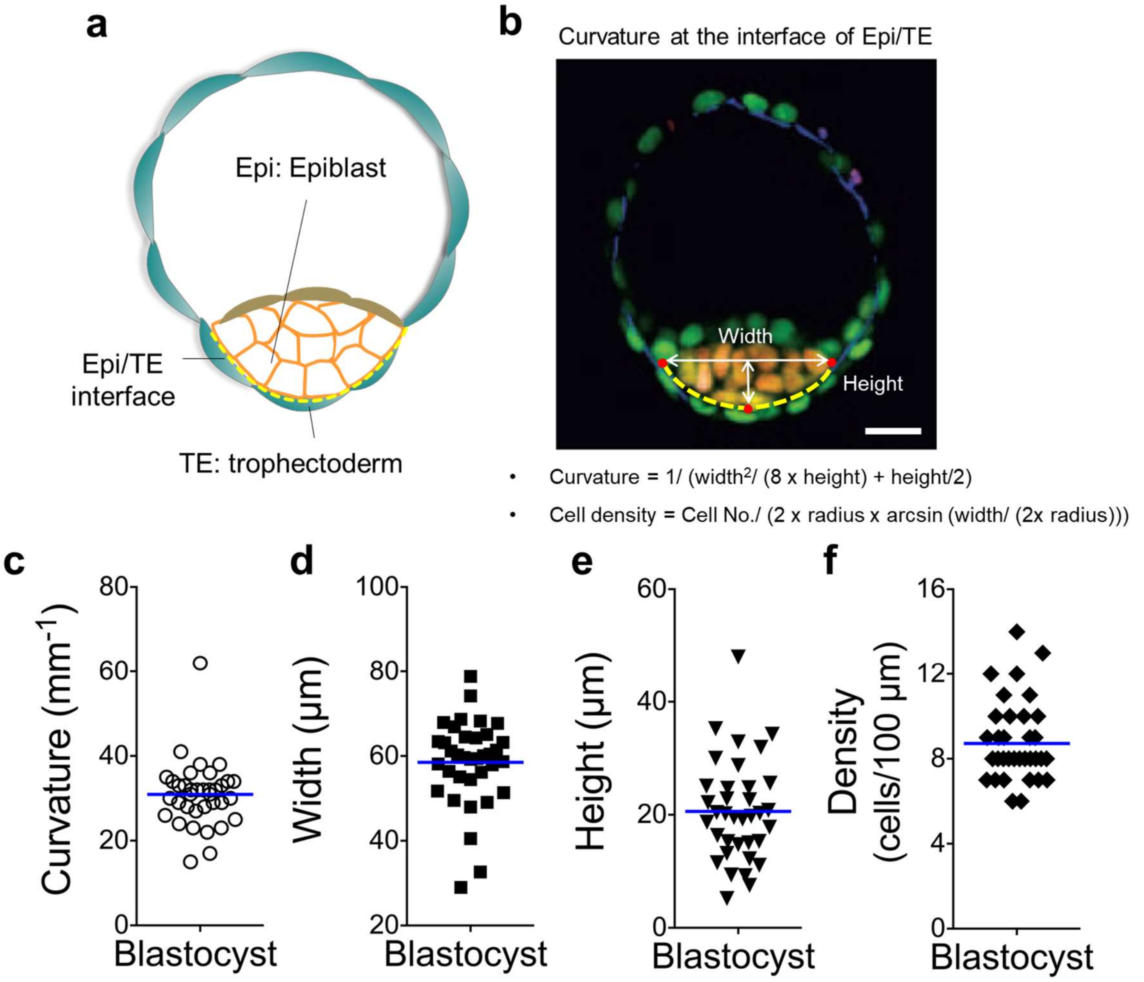

Supplementary Fig. 1. Characterization of pre-implantation blastocysts.

Schematic illustration of the methodology (a) and the representative confocal image and formulas (b) for analyzing the curvature of outer layer of nPSCs in blastocyst. (b) blastocyst image from [4]; permission was obtained. Scale bar, 20 µm. The scatter plots showing the curvature distribution (c), width (d), height (e) and density (f) of outer layer of nPSCs at Epi/TE interface in blastocysts (n = 36 biologically independent samples).

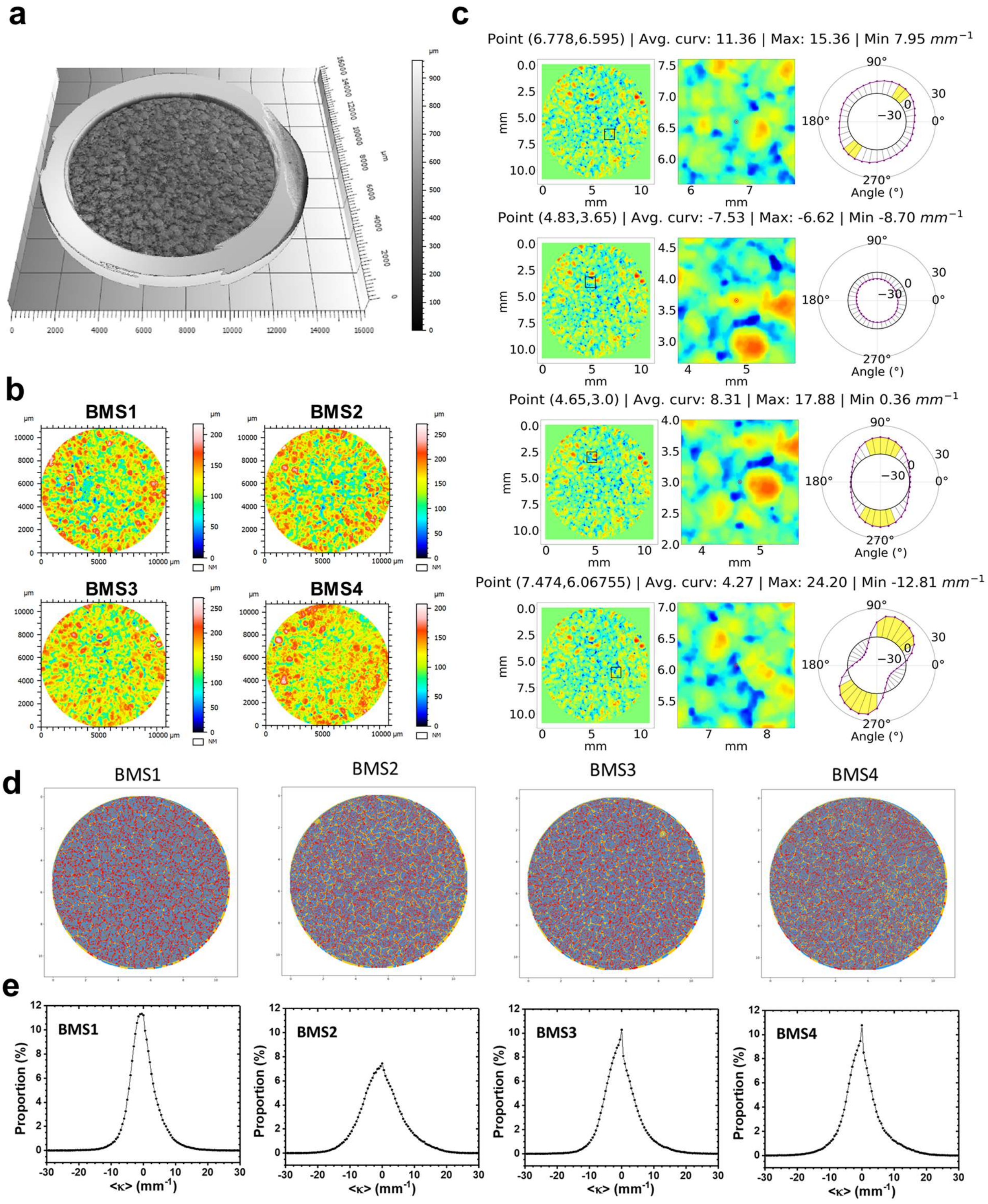

Supplementary Fig. 2. Motif and curvature distribution of BMS surfaces.

a. Reconstructed 3D image of polystyrene BMS1 based on micro-CT. **b.** Height profiles of BMS1 - 4. The total diameter is 10.8 mm. **c.** Examples of radar chart curvature plots of BMS3 using the "scaled three-point curvature" with a scale of 30 µm. The left column shows the location on the BMS3, the middle column shown a zoomed in image of the region of interest, and the right column shows the radar chart curvature, κ, plots. Here the values between BSCR 15 $mm^{-1} < \kappa < 62$ mm^{-1} are colored yellow, and the black circle marks zero curvature. **d.** Motif

maps of BMS1 – BMS4. ⟨κ⟩ Convex (⟨κ⟩ <-2.5 mm^{-1}, blue), Flat (-2.5 mm^{-1}< ⟨κ⟩ < 2.5 mm^{-1}, gray), Concave (⟨κ⟩ > 2.5 mm^{-1}, red). For the Concave motif regions we focus on the subset BSCR (15 mm^{-1}< κ < 62 mm^{-1}, yellow). **e.** Distribution of average curvature ⟨κ⟩ for the polymeric microstructured BMS1-BMS4.

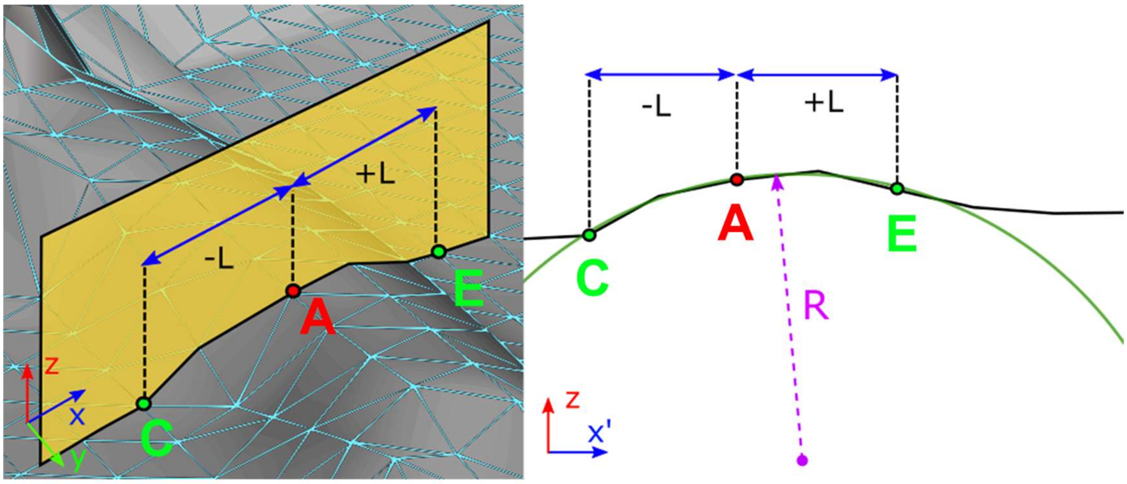

Supplementary Fig. 3. Scaled three-point approach for curvature measurement.

Schematic illustration of scaled three-point approach for curvature measurement. A plane cut (yellow) was made to measure the curvature of point A on the defined direction. Two other points, C and E, are chosen, equidistant from A in the given plane, which defines a circle with radius r in the given plane cut. The total analyzed length for the measured points is 2L = 60 μm.

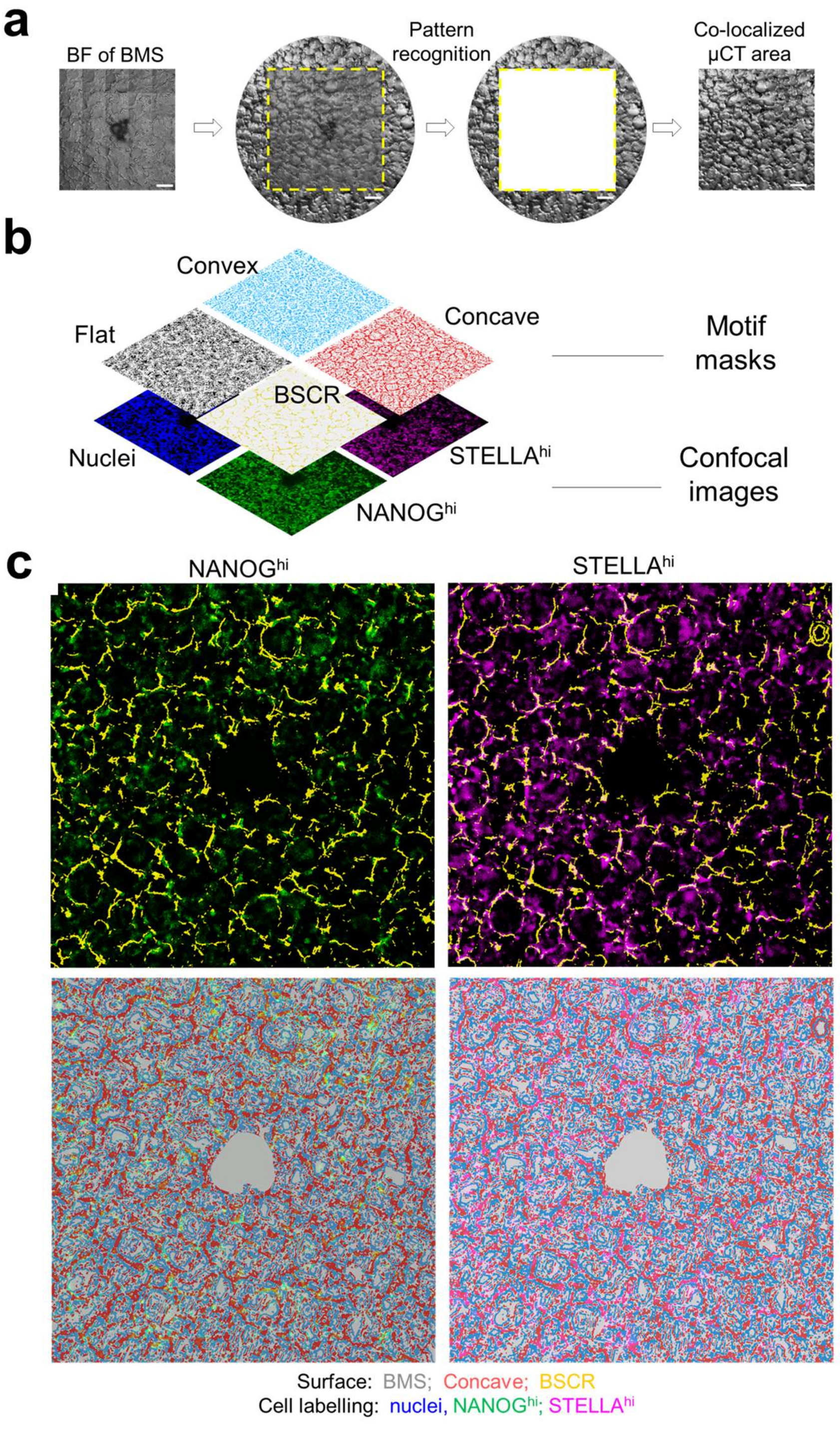

Supplementary Fig. 4. Correlative analysis integrating geometrical and biological information.

a. Co-localization of images from laser scanning confocal microscope and micro-CT. **b.** Motif masks and LSM fluorescence staining images for in situ analysis. **c.** Overlay images of NANOG^{hi} and STELLA^{hi} with different motif images. Three experiments were repeated independently. Scale bar, 500 µm.

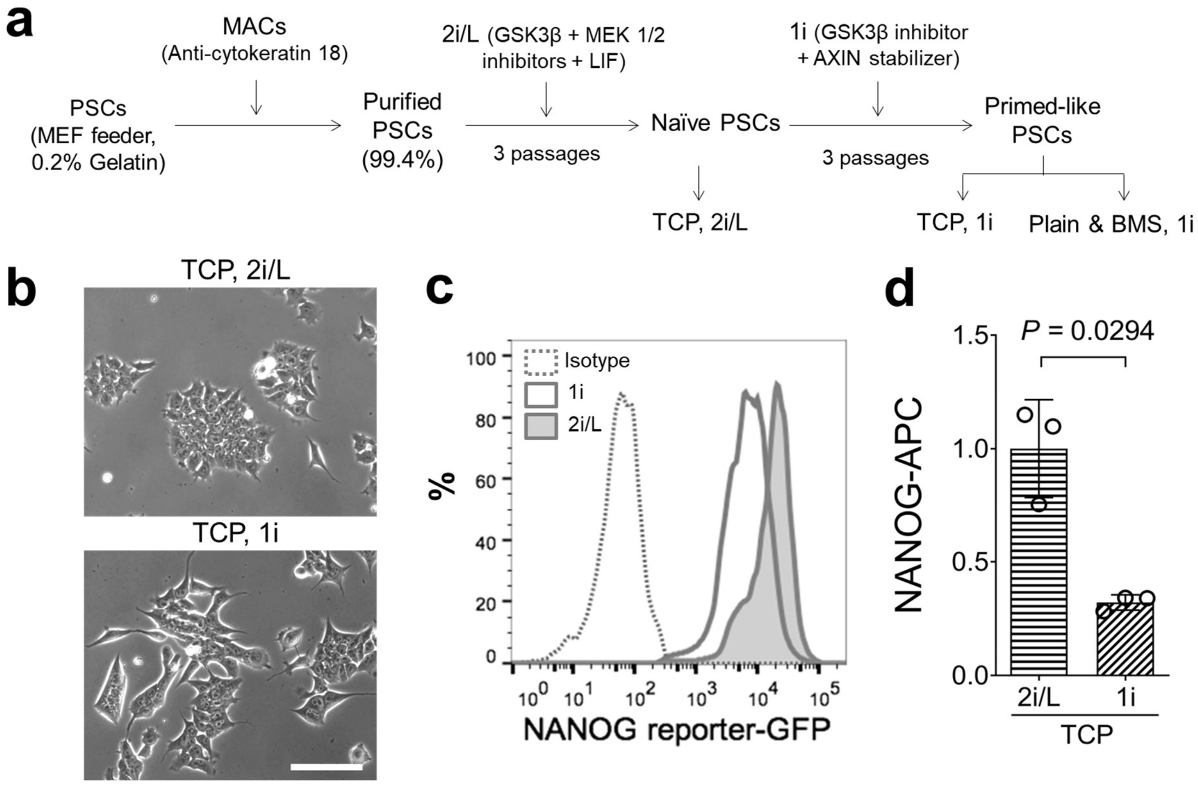

Supplementary Fig. 5. Characterization of PSCs with naïve and primed-like states.

a. Scheme of cell manipulation and gating strategy for flow cytometric analysis of MACs-based PSC purification. **b.** Morphology of naïve (in 2i/L medium) and primed-like (in 1i medium) PSCs. Scale bar, 100 µm. Flow cytometry analysis of NANOG reporter-GFP (**c**) and quantification of NANOG protein level (**d**) TCP,2i/L group was set as 1. (n=3 biologically independent samples per group; Data presented as means ± SD.; Statistical significance was calculated via two-tailed Student's t test).

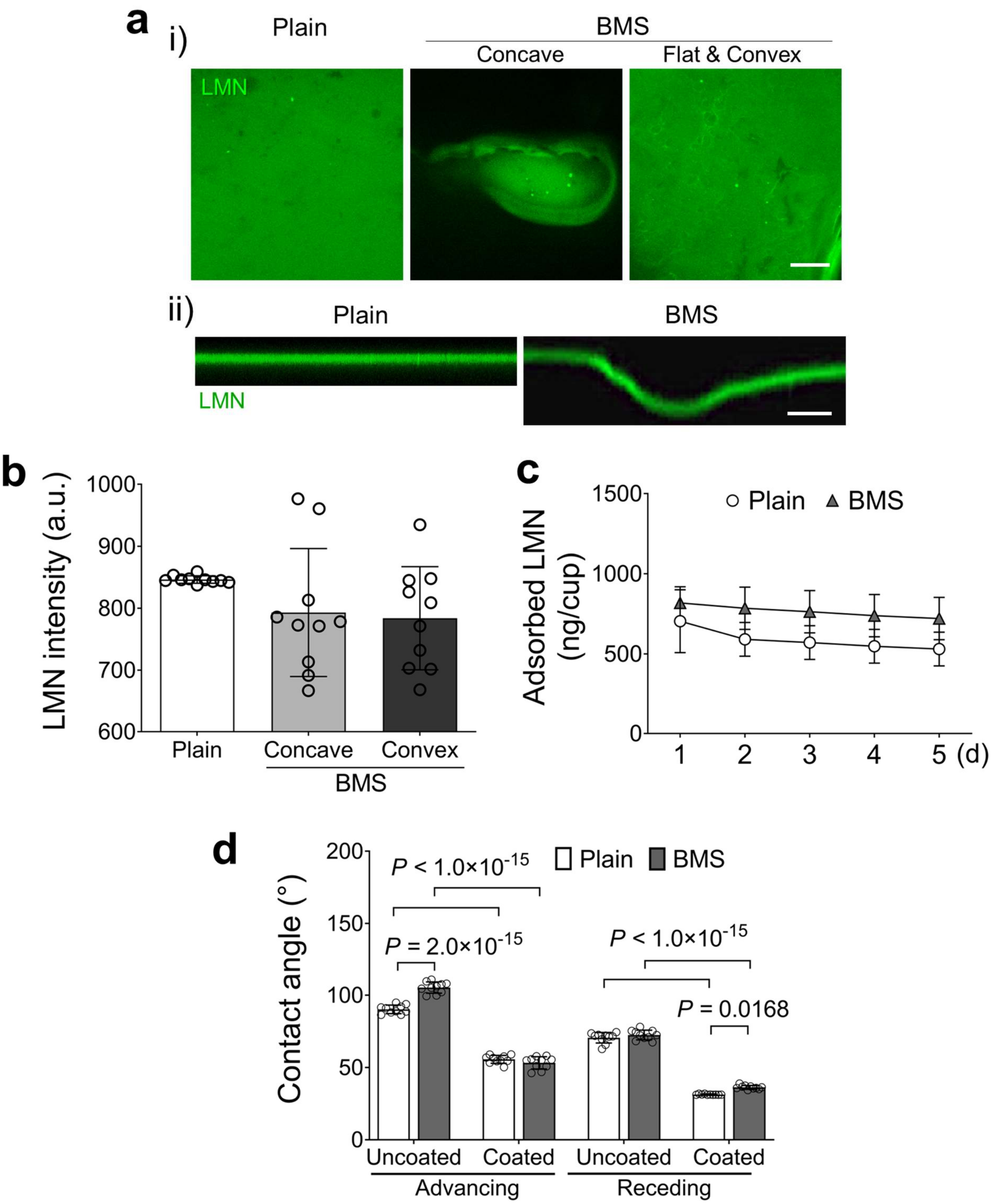

Supplementary Fig. 6. Characterization of laminin (LMN) coated Plain and BMS substrates.

a. Confocal microscopic images of top-view (i) and side-view (ii) of LMN coated Plain and BMS substrates. Scale bar, 50 μm. **b.** Quantification of LMN density in different surfaces based on the analysis on confocal images using ImageJ software (a.u.: arbitrary units) (n=10 biologically independent samples per group; Data presented as means ± SD.; Statistical significance was calculated via two-way ANOVA with Bonferroni's multiple comparisons test). **c.** Quantification of the LMN adsorption on Plain and BMS via ELISA (n=3 biologically

independent experiments; Data presented as means ± SD.; Statistical significance was calculated via one-way ANOVA with Bonferroni's multiple comparisons test). **d.** The surface wettability of Plain and BMS substrate with and without LMN determined via contact angle measurement (n=10 measurements of 5 locations of each sample. Data presented as means ± SD.; Statistical significance was calculated via two-way ANOVA with Bonferroni's multiple comparisons test).

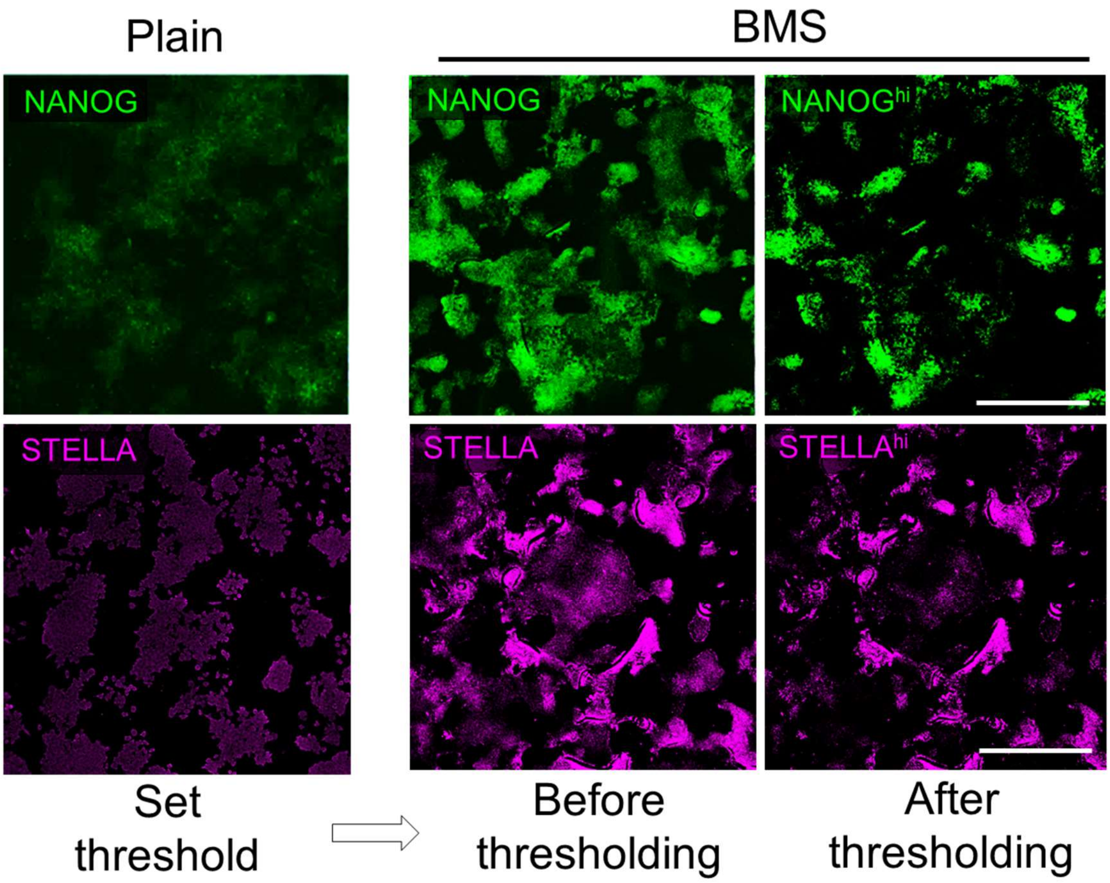

Supplementary Fig. 7. Threshold determination for the confocal images of BMS-PSCs.

The mean fluorescence intensity (MFI) of NANOG and STELLA of cells growing on the Plain surface was analyzed and used as a threshold to distinguish the different levels of fluorescence signals on BMS substrate. The fluoresce signals of NANOG and STELLA lower than the threshold were removed and the remained fluorescence was defined as "NANOG High ($NANOG^{hi}$)" and "STELLA High ($STELLA^{hi}$)" (Images represent the results from 3 independent experiments; Scale bar, 300 μm).

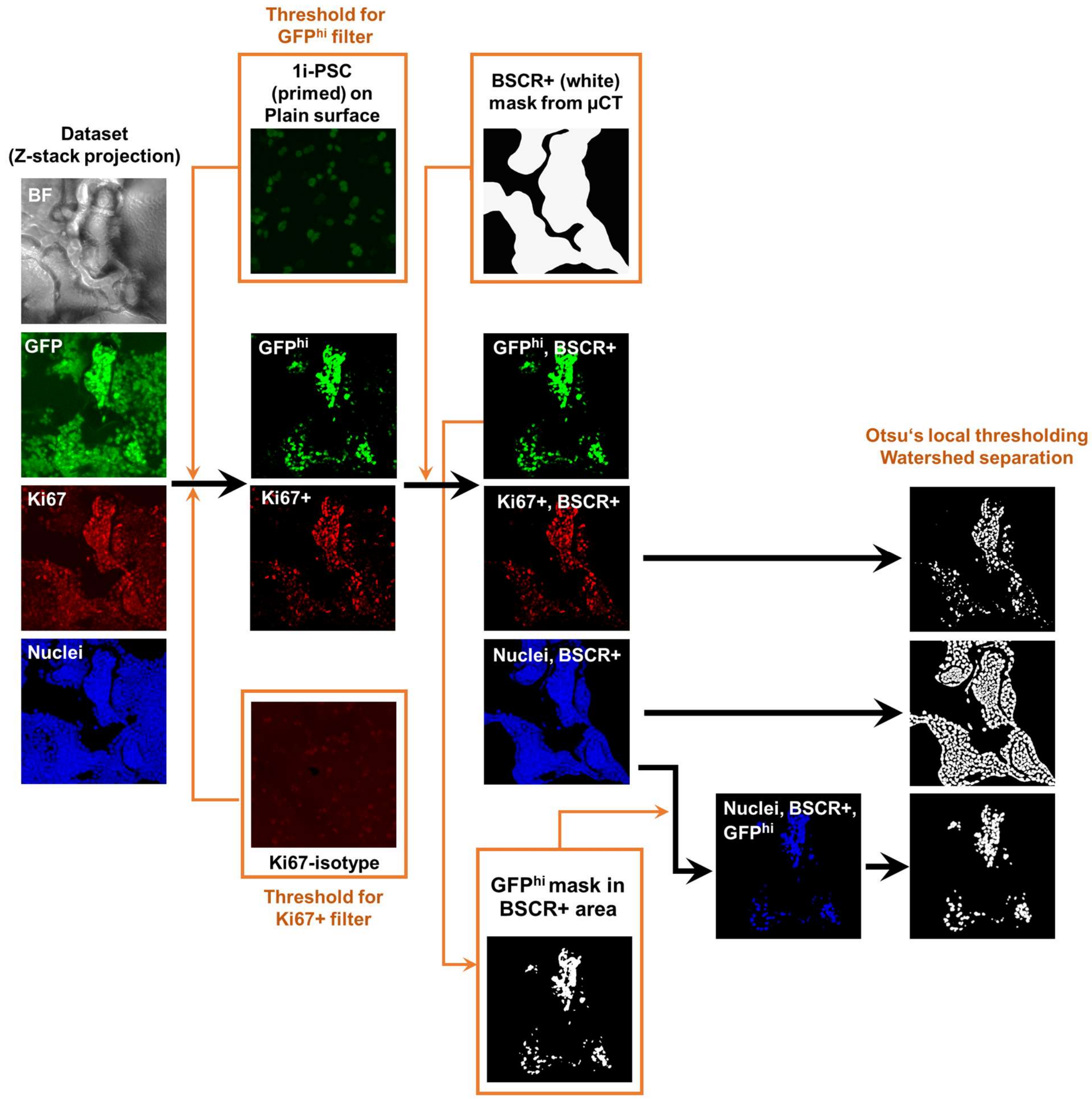

Supplementary Fig. 8. Image processing and analysis for naïvety reversion and cell proliferation.

The flowchart showed an example of analysis of Ki67 expression, GFP level and nuclei distribution of cells on BSCR+ area of a BMS substrate.

Supplementary Tables

Supplementary Table 1. Comparism of surface parameters between metal molds and BMS according to DIN EN ISO 25178

Property	Parameter	Unit	Metal molds	BMS
Arimethical mean hight of the surface	Sa	µm	19.7±0.7	20.7±2.1
Root mean square height of the surface	Sq	µm	25.2±0.7	26.1±2.0
Maximum height of peaks	Sp	µm	103±8	72.7±7.2
Maximum height of valleys	Sv	µm	68.7±8.4	105±1.0
Maximum height of the surface	Sz	µm	172±14	178±7
Skewness of height distribution	Ssk	-	0.49±0.23	-0.57±0.13
Kurtosis of height distribution	Sku	-	3.5±0.4	3.3±0.5
Root mean square gradient of the surface	Sdq		0.43±0.03	0.45±0.03
Developed area ratio	Sdr	%	8.0±0.8	8.6±1.0
Arithmetic mean peak curvature	Spc	mm^{-1}	17.4±0.9	12.1±7.5
Mean dale volume	Sdv	10^4 $µm^3$	56.2±11.4	32.5±6.3
Areal material ratio	Smr(c=0µm)	%	0.004±0.0	0.008±0.01
Inverse areal mat ratio	Smc(mc=80%)	µm	33.4±0.1	30.6±3.3
Void volume of valleys	Vvv	ml/m^2	2.40±0.36	3.66±0.19
Void volume of the core	Vvc	ml/m^2	32.5±0.7	27.7±4.1
Material volume of peaks	Vmp	ml/m^2	1.61±0.15	0.84±0.10
Material volume of core	Vmc	ml/m^2	21.3±1.3	24.1±3.4
Void volume of the core (mc=20%)	Vvc(20/80)	ml/m^2	20.8±0.8	20.7±2.9
Material volume of peaks (mc=20%)	Vmp(20/80)	ml/m^2	3.59±0.34	2.04±0.20
Material volume of core (mc=20%)	Vmc(20/80)	ml/m^2	19.3±1.5	22.9±3.1

Supplementary Table 2. Parameters of microbowls

Microbowl (μB)	Diameter of used glass beads (μm)	Microbowl diameter (μm)	Microbowl depth (μm)	Microbowl radius (μm)	Microbowl curvature κ (mm^{-1})	BSCR counts
μB-k28-BSCR 18	75	72.0 ± 1.1	25.0 ± 2.2	36.1 ± 1.3	27.7 ± 0.9	18
μB-k14-BSCR 0	150-210	155.4 ± 7.6	57.8 ± 8.5	69.5 ± 6.5	14.4 ± 1.2	0
μB-k11-BSCR 0	250	214.6 ± 12.3	83.2 ± 4.3	93.1 ± 2.7	10.7 ± 0.3	0

Supplementary Table 3. Parameters of microgrooves

Microgroove (μG)	Groove width (μm)	Interspace between grooves (μm)	Groove depth (μm)	Groove cross-sectional radius (μm)	Groove cross-sectional curvature κ (mm^{-1})	BSCR counts
μG-k32-BSCR 11	61.6 ± 0.7	34.0 ± 0.4	25.6 ± 0.8	31.3 ± 0.3	31.9 ± 0.3	11
μG-k17-BSCR 5	85.7 ± 5.4	56.1 ± 0.5	19.7 ± 1.1	58.0 ± 3.5	17.3 ± 1.1	5
μG-k7-BSCR 0	161.3 ± 4.8	33.1 ± 3.9	25.7 ± 1.6	145.6 ± 7.5	6.9 ± 0.3	0

Supplementary References

1. Verein Deutsche Ingenieure (VDI). VDI 3400. *Electrical Discharge Machining (DEM) - Definitions, processes, application*: VDI-Gesellschaft Produktion und Logistik; 1975. p. 25.

2. Dusseiller MR, Schlaepfer D, Koch M, Kroschewski R, Textor M. An inverted microcontact printing method on topographically structured polystyrene chips for arrayed micro-3-D culturing of single cells. *Biomaterials* 2005, **26**(29)**:** 5917-5925.

3. Kim H, Wu J, Ye S, Tai CI, Zhou X, Yan H*, et al.* Modulation of beta-catenin function maintains mouse epiblast stem cell and human embryonic stem cell self-renewal. *Nat Commun* 2013, **4:** 2403.

4. Strumpf D, Mao CA, Yamanaka Y, Ralston A, Chawengsaksophak K, Beck F*, et al.* Cdx2 is required for correct cell fate specification and differentiation of trophectoderm in the mouse blastocyst. *Development* 2005, **132**(9)**:** 2093-2102.
