# Supplementary material for: Substrates mimicking the blastocyst geometry revert pluripotent stem cell to naivety
Source: Nat Mater. 2024 Aug 12;23(12):1748–58. doi: 10.1038/s41563-024-01971-4 (PMC11599042; doi:10.1038/s41563-024-01971-4)
Supplement: Supplementary file 2 — Reporting Summary [file 41563_2024_1971_MOESM2_ESM.pdf]

nature portfolio

Corresponding author(s): Prof. Dr. Andreas Lendlein, Prof. Dr. Nan Ma

Last updated by author(s): May 15, 2024

Reporting Summary

Nature Portfolio wishes to improve the reproducibility of the work that we publish. This form provides structure for consistency and transparency in reporting. For further information on Nature Portfolio policies, see our Editorial Policies and the Editorial Policy Checklist.

Statistics

For all statistical analyses, confirm that the following items are present in the figure legend, table legend, main text, or Methods section.

n/a	Confirmed	
☐	☒	The exact sample size (*n*) for each experimental group/condition, given as a discrete number and unit of measurement
☐	☒	A statement on whether measurements were taken from distinct samples or whether the same sample was measured repeatedly
☐	☒	The statistical test(s) used AND whether they are one- or two-sided<br>*Only common tests should be described solely by name; describe more complex techniques in the Methods section.*
☒	☐	A description of all covariates tested
☐	☒	A description of any assumptions or corrections, such as tests of normality and adjustment for multiple comparisons
☐	☒	A full description of the statistical parameters including central tendency (e.g. means) or other basic estimates (e.g. regression coefficient) AND variation (e.g. standard deviation) or associated estimates of uncertainty (e.g. confidence intervals)
☐	☒	For null hypothesis testing, the test statistic (e.g. *F*, *t*, *r*) with confidence intervals, effect sizes, degrees of freedom and *P* value noted<br>*Give P values as exact values whenever suitable.*
☒	☐	For Bayesian analysis, information on the choice of priors and Markov chain Monte Carlo settings
☒	☐	For hierarchical and complex designs, identification of the appropriate level for tests and full reporting of outcomes
☒	☐	Estimates of effect sizes (e.g. Cohen's *d*, Pearson's *r*), indicating how they were calculated

Our web collection on statistics for biologists contains articles on many of the points above.

Software and code

Policy information about availability of computer code

Data collection	ZEN 2012 and Zen 2.3(Carl Zeiss Microscopy GmbH); MACSQuant (Miltenyi Biotec.); Xcellence/cell^R (Olympus); Odyssey Infrared Imaging software ver 3.0.25 (LI-COR Biosciences); Magellan software ver 7.4 (Tecan Group Ltd.)
Data analysis	MAVI (Fraunhofer Society); MountainsMap (Digital Surf, France); ImageJ ver 2.0.0-rc-65/1.52b (National Institutes of Health); ModFit LT (Verity Software House); Flowjo ver 10.4 (Tree Star Inc.); Photoshop CS6 (Adobe Systems); Prism GraphPad 8.0 (GraphPad Software), Origin (Originlab), Matlab (Mathworks), Spyder (Python 3.8, Anaconda Inc.). The custom code written for this manuscript uses Python 3.8 and the accompanying packages numpy, numpy-stl, matplotlib, scipy, and pandas were used to analyze the different substrates mentioned in the paper. The code scripts are available in the Zenodo repository via the following link: https://doi.org/10.5281/zenodo.10732261.

For manuscripts utilizing custom algorithms or software that are central to the research but not yet described in published literature, software must be made available to editors and reviewers. We strongly encourage code deposition in a community repository (e.g. GitHub). See the Nature Portfolio guidelines for submitting code & software for further information.

Data

Policy information about availability of data

All manuscripts must include a data availability statement. This statement should provide the following information, where applicable:

- Accession codes, unique identifiers, or web links for publicly available datasets
- A description of any restrictions on data availability
- For clinical datasets or third party data, please ensure that the statement adheres to our policy

All data supporting the findings of this study are available within the article and its supplementary information files. Source data can be found on Figshare at https://doi.org/10.6084/m9.figshare.25827706. Source data are provided with this paper.

Research involving human participants, their data, or biological material

Policy information about studies with human participants or human data. See also policy information about sex, gender (identity/presentation), and sexual orientation and race, ethnicity and racism.

Reporting on sex and gender	not applicable
Reporting on race, ethnicity, or other socially relevant groupings	not applicable
Population characteristics	not applicable
Recruitment	not applicable
Ethics oversight	not applicable

Note that full information on the approval of the study protocol must also be provided in the manuscript.

Field-specific reporting

Please select the one below that is the best fit for your research. If you are not sure, read the appropriate sections before making your selection.

☒ Life sciences ☐ Behavioural & social sciences ☐ Ecological, evolutionary & environmental sciences

For a reference copy of the document with all sections, see nature.com/documents/nr-reporting-summary-flat.pdf

Life sciences study design

All studies must disclose on these points even when the disclosure is negative.

Sample size	Experiments performed in the study were conducted with independent experiments/multiple biological replicates (including separate batches/plates of cells, cell lysates and immunodeficient mice) across Plain and BMS groups. Sample sizes are determined by data saturation, when no new information emerges from additional biologically independent samples and experiments. Based on the previous experiences and similar study performed from other researchers (Reference: Stem Cell Res. 2013 Jan;10(1):118-31. and Nat Mater. 2016 Mar;15(3):344-52.), Three biologically independent experiments were considered as sufficient number since they led to similar results.
Data exclusions	No data exclusion was performed in the study.
Replication	To verify the reproducibility of the teratoma formation results shown in Fig.6j,k, we repeated the teratoma test using different cell concentration and the similar results were obtained, which supporting the reproducibility of higher teratoma formation (pluripotency) potential using primed-like mouse iPSCs that preconditioned with BMS substrates. For other analysis including quantification of naïve pluripotency associated markers, E-cad, FAK, YAP, histone H3 modification at naïve gene loci, cell death, migration, cell cycle sub-phases, cell expansion, at least 3 biologically independent experiments across Plain and BMS. All attempts at replication were successful for those experiments.
Randomization	The Plain and BMS preconditioning groups were randomly numbered as group A and B or C for each teratoma experiment. At least one week prior to iPSC injection, Three of each 8-week old NSG immunodeficient male mice that maintained in EPO GmbH company were randomly divided into two groups. And the technicians who performed the injection and the following measurements on teratoma volume were blinded to group design. All 18 mice were randomly allocated into Plain and BMS groups. The Plain and BMS polymeric inserts from different batches that fit cell culture plate were taken and placed in the culture wells prior to cell seeding and cells were seeded randomly to each polymeric inserts. The BMS polymeric inserts were created by 4 microstructured metal molds and selected randomly for all the biological analysis. However, no particular randomization method was used.
Blinding	Teratoma studies using animals were performed by nonblinded and blinded personnel in a random fashion. The investigators were not blinded to the allocation during the in vitro experiments, but the researchers who conducted the analysis were blinded. Since we use different

types of cell culture substrates, including BMS and Plain, which can be visually distinguished, it is challenging to keep the operators blind regarding group settings.

Reporting for specific materials, systems and methods

We require information from authors about some types of materials, experimental systems and methods used in many studies. Here, indicate whether each material, system or method listed is relevant to your study. If you are not sure if a list item applies to your research, read the appropriate section before selecting a response.

Materials & experimental systems

n/a	Involved in the study
☐	☒ Antibodies
☐	☒ Eukaryotic cell lines
☒	☐ Palaeontology and archaeology
☐	☒ Animals and other organisms
☒	☐ Clinical data
☒	☐ Dual use research of concern
☒	☐ Plants

Methods

n/a	Involved in the study
☒	☐ ChIP-seq
☐	☒ Flow cytometry
☒	☐ MRI-based neuroimaging

Antibodies

Antibodies used

For immunocytochemistry staining:
anti-NANOG (rabbit polyclonal; 1:10; Cat. No.: PA1-41577; Thermo Fisher Scientific Inc.),
anti-STELLA (mouse monoclonal; 3H5.2; 1:50; MAB4388; Merck KGaA),
anti-ki67 (rabbit monoclonal; D3B5; 1:400; Cat. No.: 9129S; New England Biolabs GmbH),
Rabbit IgG Isotype Control (1:400; Cat. No.: 31235; Thermo Fisher Scientific Inc.),
anti-E-Cadherin-PE (rabbit monoclonal 24E10; 1:50; Cat. No.: 7559; New England Biolabs GmbH),
anti-YAP (rabbit monoclonal; D8H1X; 1:100; Cat. No.: 14074; New England Biolabs GmbH),
anti-Laminin (rabbit monoclonal; 1:100; Cat.No.: ab11575; Abcam),
anti-phospho-myosin light chain 2 (S19) (mouse monoclonal; 1:100; Cat. No.: 3675; New England Biolabs GmbH),
StainAlive DyLight 488 conjugated anti-SSEA1 antibody (mouse monoclonal; MC-480; 1:100; Cat. No.: 09-0067; Stemgent, Inc.),
anti-rabbit IgG (H+L) -Alexa fluor 488 secondary antibody (goat polyclonal 1:800; Cat. No.: A-11070; Thermo Fisher Scientific Inc.),
anti-rabbit IgG (H+L) -Alexa fluor 633 secondary antibody (goat polyclonal 1:800; Cat. No.: A-21071; Thermo Fisher Scientific Inc.).
anti-mouse IgG (H+L) -Alexa fluor 647 secondary antibody (goat polyclonal 1:500; Cat. No.: 4410S; New England Biolabs GmbH.),
anti-rabbit IgG (H+L) -Alexa fluor 647 secondary antibody (goat polyclonal 1:500; Cat. No.: 4414S; New England Biolabs GmbH.).

For flow cytometry:
anti-SSEA1-PE (mouse monoclonal MC-480; 1:50; Cat. No.: MA1-022-PE; Thermo Fisher Scientific Inc.),
anti-SSEA-4- Alexa fluor 647 (mouse monoclonal MC813-70; 1:10; Cat. No.: 560219; BD Biosciences),
anti-E-Cadherin-PE (rabbit monoclonal 24E10; 1:50; Cat. No.: 7559; New England Biolabs GmbH),
anti-NANOG-APC (REAfinity recombinant human IgG; REA297; 1:11; Cat. No.: 130-104-480; Miltenyi Biotec.),
anti-OCT4-PE (REAfinity recombinant human IgG; REA622; 1:11; Cat. No.: 130-109-716 Miltenyi Biotec.),
anti-STELLA (mouse monoclonal; 3H5.2; 1:50; MAB4388; Merck KGaA),
anti-TBX3 (rabbit polyclonal; 1:200; Cat. No.: 42-4800; Thermo Fisher Scientific Inc.)
anti-ZIC2 (rabbit monoclonal; EPR7790; 1:600; Cat. No.: ab150404; abcam),
anti-phospho-YAP (S127) (rabbit monoclonal; D9W2I; 1:50; Cat. No.: 13008; New England Biolabs GmbH),
anti-YAP-Alexa fluor 647 (rabbit monoclonal; D8H1X; 1:50; Cat. No.: 38707; New England Biolabs GmbH),
anti-phospho-AMOTL1 (S262) (rabbit polyclonal; 1:100; pab0956-P; Covalab Biotechnology),
anti-phospho-myosin light chain 2 (S19) (mouse monoclonal; 1:100; Cat. No.: 3675; New England Biolabs GmbH),
anti-CD7-APC (REAfinity recombinant human IgG; REA622; 1:50; Cat. No.: 130-124-941; Miltenyi Biotec.),
anti-CD24-APC (REAfinity recombinant human IgG; REA832; 1:50; Cat. No.: 130-112-846; Miltenyi Biotec.),
anti-CD57-FITC (mouse IgM monoclonal; 1:50; Cat. No.: 130-122-935; Miltenyi Biotec.),
anti-CD90-FITC (REAfinity recombinant human IgG; 1:50; Cat. No.: 130-114-901; Miltenyi Biotec.),
anti-Cytokeratin 18 antibody (rabbit monoclonal; EPR17347; 1:120; Cat. No.: ab181597; Abcam),
REA Control-APC (REAfinity recombinant human IgG REA293; 1:50; Cat. No.: 130-113-446; Miltenyi Biotec.),
REA Control-PE (REAfinity recombinant human IgG REA293; 1:50; Cat. No.: 130-113-450; Miltenyi Biotec.),
anti-mouse IgG (H+L)-Alexa fluor 647 (goat polyclonal; 1:500; Cat. No.: 4410; New England Biolabs GmbH),
anti-rabbit IgG (H+L)-Alexa fluor 647 (goat polyclonal; 1:500; Cat. No.: 4414; New England Biolabs GmbH).

For immunoblotting:
anti-E-cadherin (mouse monoclonal; 7H12; 1:200; Cat. No.: MA5-15711; Thermo Fisher Scientific Inc.),
anti-phospho-YAP (S127) (rabbit monoclonal; D9W2I; 1:1000; Cat. No.: 13008; New England Biolabs GmbH),
anti-YAP (rabbit monoclonal; D8H1X; 1:100; Cat. No.: 14074; New England Biolabs GmbH),
anti-GAPDH (rabbit monoclonal; D16H11; 1:1000; Cat. No.: 5174; New England Biolabs).

For ChIP-PCR:
anti-histone H3 (rabbit monoclonal; D2B12; 1:50; Cat. No.: 4620; New England Biolabs GmbH),
anti-H3K27me3 (rabbit monoclonal; C36B11; 1:50; Cat. No.: 9733; New England Biolabs GmbH),

anti-H3K4me3 (rabbit monoclonal; C42D8; 1:50; Cat. No.: 9751; New England Biolabs GmbH),
Normal rabbit IgG (rabbit polyclonal; 1:50; Cat. No.: 2729; New England Biolabs GmbH).

Validation	Isotype antibodies were applied accordingly in each flow cytometric experiment to avoid the non-specific binding of target antigens. Positive samples were used for validating the specificity of target antibodies. Blocking solution (PBS containing 0.5 wt% BSA) was applied for all flow cytometry assays. PBS containing 3 wt% BSA was used as blocking reagent for all immunostaining experiments. For immunoblotting, Licor blocking buffer was used. Target marker positive cell Lysates were purchased used and series dilution was applied for testing the linear range for immunoblotting. Application of secondary antibody without primary antibody was also involved for avoiding non-specific binding of secondary antibodies. Primary antibodies were validated by using positive control samples (e.g. in fig.2a, 5c-d, extended data fig.3-4, 7c, 8b-d, and supplementary fig.5c-d).

Eukaryotic cell lines

Policy information about cell lines and Sex and Gender in Research

Cell line source(s)	The mouse iPSC cell line PhiC31 (Cat.No.: SC211A-1, BioCat GmbH, Germany) was reprogrammed from C57BL/6 embryonic fibroblasts (MEFs) with a plasmid encoding Oct4, Sox2, Klf4, and c-Myc were used for assessing the reversion to naïvety.<br><br>The mouse iPSC cell line with NANOG-GFP reporter (iPS-MEF-Ng-492B-4 cells, Cat.No.: APS0004, CiRA, Kyoto University, Japan) were used for living cell expansion tracking. This cell line was reprogrammed from mouse embryonic fibroblasts without retrovirus vector and no integration of exogene transfection.<br><br>BIHi001-A human iPSC cell line was a gift from the Stem Cell Core Facility, Berlin Institute of Health, Germany. This exogene integration-free cell line was generated using Sendai virus vectors.<br><br>The mouse embryonic fibroblasts feeder cells (Cat.No.: CBA-310-CB, BioCat GmbH, Germany) were used for initial recovering of both aforementioned mouse iPSC cell lines.<br><br>The irradiated CF1 mouse embryonic fibroblasts (Cat.No.: A34181, ThermoFisher Scientific) was used for naïve pluripotency reversion of BIHi001-A human iPSCs.
Authentication	The providers validated the cell lines. For PhiC31: morphology, Immunostaining of stemness markers, and alkaline phosphatase activity. For iPS-MEF-Ng-492B-4 cell line: morphology, gene expression, GFP reporter expression, teratoma, and chimeric competency assay. For BIHi001-A: morphology, gene expression array, Immunostaining and flow cytometry for stemness markers, PluriTest with ILLUMINA Microarray to confirm pluripotency, Karyotyping for genomic stability. Detailed information of PhiC31 (Cat.No.: SC211A-1, BioCat GmbH, Germany) is available online https://www.biocat.com/bc/pdf/PAC_SC211A-1_140210-01_with_safety_notice.pdf; Detailed information of iPS-MEF-Ng-492B-4 cells (Cat.No.: APS0004, CiRA, Kyoto University, Japanis) is available online https://cellbank.brc.riken.jp/cell_bank/CellInfo/?cellNo=APS0004&lang=En; Detailed information BIHi001-A human iPSC cell line (BIH, Germany) is available in the hPSCreg database (http://hpscreg.eu/cell-line/BIHi001-A).
Mycoplasma contamination	All iPSCs and MEF feeder cells applied for experiments were detected negative for mycoplasma using MycoFluor Mycoplasma Detection Kit (Thermo Fisher Scientific, Germany).
Commonly misidentified lines<br>(See ICLAC register)	No commonly misidentified cell lines were used.

Animals and other research organisms

Policy information about studies involving animals; ARRIVE guidelines recommended for reporting animal research, and Sex and Gender in Research

Laboratory animals	NSG immunodeficient mice, male, 8-week old were used for teratoma formation study. The animals were maintained under a 12-hour light/dark cycle at a housing temperature of 22°C ± 1°C, 50% relative humidity, and access to autoclaved food, bedding material, and tap water ad libitum.
Wild animals	The study did not involve wild animals.
Reporting on sex	The findings apply to male NSG mice in this study.
Field-collected samples	The study did not involve the samples collected from the field.
Ethics oversight	The animal experiment was performed by EPO GmbH (Experimental Pharmacology and Oncology, Berlin-Buch) and carried out in accordance with the of the German Animal Protection Law and approved by the local responsible authorities. EPO complies to the EU guideline "European convention for the protection of vertebrate animals used for experimental and other scientific purposes. (EST 123)". The male NSG immunodeficient mice, 8-week old were handled in EPO GmbH according to the "Regulation on the protection of experimental scientific purposes or other Purposes used animals". Compliance with the above rules and regulations is monitored by the Landesamt fuer Gesundheit und Soziales (LAGeSo) which is the responsible regulatory authority monitoring the animal husbandry based on the German Animal Welfare Act. Approval was given after careful inspection of the site including bedding, feeding & water, ventilation, temperature, and humidity, cleaning and hygiene concepts.

Note that full information on the approval of the study protocol must also be provided in the manuscript.

Flow Cytometry

Plots

Confirm that:

☒ The axis labels state the marker and fluorochrome used (e.g. CD4-FITC).

☒ The axis scales are clearly visible. Include numbers along axes only for bottom left plot of group (a 'group' is an analysis of identical markers).

☒ All plots are contour plots with outliers or pseudocolor plots.

☒ A numerical value for number of cells or percentage (with statistics) is provided.

Methodology

Sample preparation	Single cells from mouse iPSC cell line PhiC31 (Cat.No.: SC211A-1, BioCat GmbH, Germany), iPS-MEF-Ng-492B-4 cells (Cat.No.: APS0004, CiRA, Kyoto University, Japanis) and BIHi001-A human iPSC cell line (BIH, Germany) were freshly detached and harvested from culture substrates at indicated time points using Accutase (Merck KGaA), then immediately processed for flow cytometry analysis of surface markers or fixed and permeabilized for analyzing of intracellular proteins and cell cycle. More detailed protocols can be found in Methods and Supplementary methods sections.
Instrument	MACSQuant Analyzer 10 Flow Cytometers (Miltenyi Biotec.)
Software	Flow cytometry data were collected using MACSQuant software (Miltenyi Biotec.). Analysis was performed using Flowjo ver.10.4 software (Tree Star Inc.). Cell cycle was analyzed using ModFit LT ver.4.0.5 software (Verity Software House).
Cell population abundance	Cells harvested from Plain and BMS substrates were freshly collected and used directly for flow cytometry analysis without any additional abundance or sorting process for special cell population. Before adapting mouse iPSCs to 2i/L or 1i culture conditions, cells grown on MEF feeders were selected by using Feeder removal kit (Miltenyi Biotec.) and then purified by anti-cytokeratin 18 magnetic beads (Miltenyi Biotec.). The cytokeratin 18-negative fraction passing through the magnetic column were collected and validated by flow cytometry analysis using anti-cytokeratin 18 antibody (Abcam).
Gating strategy	For Immunophenotyping, The cell populations were gated based on the forward (FSC) and side scatter (SSC) and the following exclusion of doublet using FSC-A/FSC-H. The negative and positive fractions were separated by define the gate of negative population using isotype control.

☒ Tick this box to confirm that a figure exemplifying the gating strategy is provided in the Supplementary Information.
