# Supplementary figures and images for: Substrates mimicking the blastocyst geometry revert pluripotent stem cell to naivety
Source: Nat Mater. 2024 Aug 12;23(12):1748–58. doi: 10.1038/s41563-024-01971-4 (PMC11599042; doi:10.1038/s41563-024-01971-4)

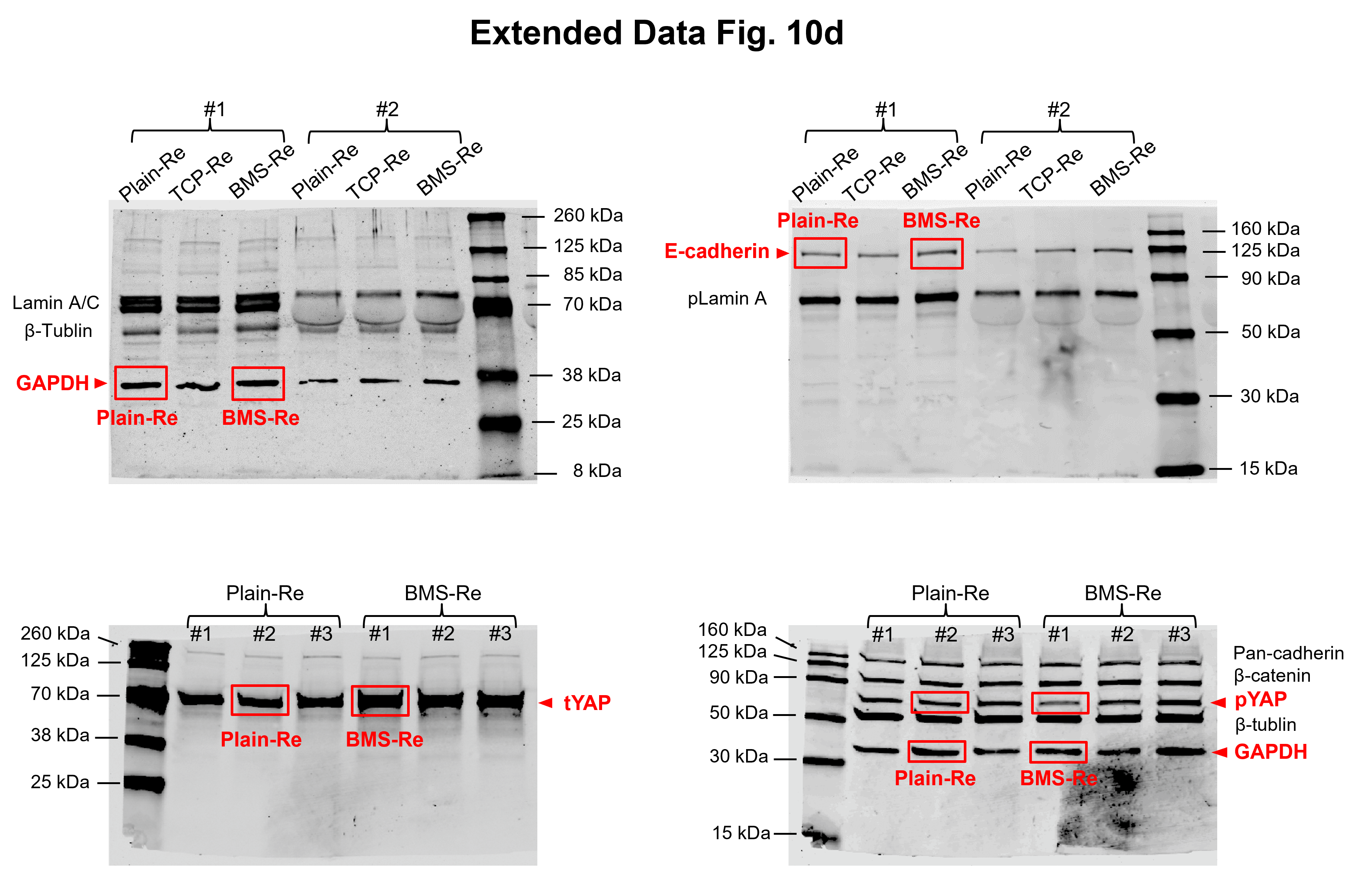

Supplement: Supplementary file 19 — Unprocessed western blots. [file 41563_2024_1971_MOESM19_ESM.tif]
